# Heavy Metal Exposure Influences Double Strand Break DNA Repair Outcomes

**DOI:** 10.1371/journal.pone.0151367

**Published:** 2016-03-11

**Authors:** Maria E. Morales, Rebecca S. Derbes, Catherine M. Ade, Jonathan C. Ortego, Jeremy Stark, Prescott L. Deininger, Astrid M. Roy-Engel

**Affiliations:** 1 Department of Epidemiology and Tulane Cancer Center, and Tulane University Health Sciences Center, 1430 Tulane Ave., New Orleans, LA 70112, United States of America; 2 Department of Cellular and Molecular Biology, Tulane University, 6400 Freret Street, New Orleans, LA 70118, United States of America; 3 Department of Radiation Biology, Beckman Research Institute of the City of Hope, 1500 E Duarte Rd., Duarte, CA 91010, United States of America; CNR, ITALY

## Abstract

Heavy metals such as cadmium, arsenic and nickel are classified as carcinogens. Although the precise mechanism of carcinogenesis is undefined, heavy metal exposure can contribute to genetic damage by inducing double strand breaks (DSBs) as well as inhibiting critical proteins from different DNA repair pathways. Here we take advantage of two previously published culture assay systems developed to address mechanistic aspects of DNA repair to evaluate the effects of heavy metal exposures on competing DNA repair outcomes. Our results demonstrate that exposure to heavy metals significantly alters how cells repair double strand breaks. The effects observed are both specific to the particular metal and dose dependent. Low doses of NiCl_2_ favored resolution of DSBs through homologous recombination (HR) and single strand annealing (SSA), which were inhibited by higher NiCl_2_ doses. In contrast, cells exposed to arsenic trioxide preferentially repaired using the “error prone” non-homologous end joining (alt-NHEJ) while inhibiting repair by HR. In addition, we determined that low doses of nickel and cadmium contributed to an increase in mutagenic recombination-mediated by Alu elements, the most numerous family of repetitive elements in humans. Sequence verification confirmed that the majority of the genetic deletions were the result of Alu-mediated non-allelic recombination events that predominantly arose from repair by SSA. All heavy metals showed a shift in the outcomes of alt-NHEJ repair with a significant increase of non-templated sequence insertions at the DSB repair site. Our data suggest that exposure to heavy metals will alter the choice of DNA repair pathway changing the genetic outcome of DSBs repair.

## Introduction

Cells possess a collection of proteins and enzymes dedicated to the maintenance of DNA that work through a complex group of DNA repair pathways. DNA is consistently damaged by both endogenous processes and external insults, making these pathways critical to cell survival. One of the more cytotoxic lesions is the introduction of double strand breaks (DSBs) to the DNA helix which results in cell death if not repaired. Mammalian cells primarily utilize two broad classes of DSB repair: homologous recombination (HR) and non-homologous end joining (NHEJ) (reviewed in [1,2]). Defects in these repair pathways, or misrepair of DSBs, cause genome instability that may ultimately result in cancer [3,4]. Numerous factors affect the decision to repair a DSB via these pathways and accumulating evidence suggests these major repair pathways both collaborate and compete with one another at DSB sites to facilitate efficient repair and promote genomic integrity [5].

Environmental exposure to heavy metals not only provides a source of DNA damage due to the induction of reactive oxygen species but also introduces cellular changes that can influence the competitive balance between repair mechanisms, altering the outcome of the DSB repair process. The ability of environmental insults to skew the DNA repair balance to favor “error-prone” outcomes should be considered when assessing exposures that contribute to an increased risk for onset of disease. Those heavy metals favoring error-prone repair of DSBs would contribute to the propagation of mutated DNA and possibly increased cancer risk. We evaluated the effect of three different heavy metals, arsenic trioxide and the soluble forms of cadmium and nickel (CdCl_2_, NiCl_2_), on DSB repair outcomes.

Three reasons guided our selection of heavy metals for evaluation. First, exposure to these heavy metals has been associated with adverse effects on human health [6]. Although the effects of heavy metals are highly varied and depend on the chemical form of each heavy metal, a clear correlation between exposure and cancer onset has been established contributing to their classification as carcinogens (reviewed in [7]). In addition, the selected heavy metals are included in the priority list of hazardous substances from the Agency for Toxic Substances and Disease Registry (ATSDR) (http://www.atsdr.cdc.gov/spl/index.html) with the following rankings: arsenic #1, cadmium #7, and nickel #57. Second, the mechanism by which these heavy metals induce cancer is unclear and likely complex. Some heavy metals have been of particular interest because of their limited ability to induce direct damage to DNA generating negative results when using some of the standard genotoxicity assays. For example, exposures to soluble cadmium are not mutagenic in bacterial assays [8,9]. These types of observations have led to the proposal that in some cases heavy-metal induced mutations are not likely due to direct DNA damage, but instead suggestive of an indirect mechanism, such as DNA repair inhibition [7]. Third, the three heavy metals selected for evaluation have been shown to inhibit individual DNA repair proteins from specific pathways. Cadmium is reported to inhibit nucleotide excision repair (NER) [10,11], non-homologous end-joining (NHEJ) [12], base excision repair (BER) [13] and mismatch repair (MMR) [12]. Both nickel and cadmium exposures have been shown to alter the zinc binding domain of XPA affecting function causing a general inhibition of NER performance [14,15]. In addition, cadmium further diminishes NER response by decreasing the amount of nuclear XPC and repressing ERCC1 expression [16] diminishing NER response [15]. Arsenic (arsenite) has been shown to inhibit Poly(ADP-ribose) polymerase-1 (PARP-1), which plays an important role in the DNA break repair processes [17]. Lymphocytes from individuals exposed to arsenic showed a repression of ERCC1, XPB, and XPF [18]. Nickel has been shown to alter DNA repair pathways by modifying the epigenetic regulation of genes [19]. For example, gene expression profiles of blood cells from individuals with high exposure to nickel showed that 29 out of 31 DNA repair genes were repressed [20]. These types of observations have led to the proposal that inhibition of DNA repair proteins plays an important role in metal-induced pathogenesis [7]. Overall, heavy metals have the potential to hinder the ability of cells to properly manage insults to the DNA integrity.

Multiple assays have been used to evaluate the effects of heavy metals on DNA damage and repair pathways such as comet assays, quantitation of chromosome aberrations, detection of sister chromatid exchange, and micronucleus tests. Although these assays provide important information, most of them are uni-faceted and offer little insight on how exposures affect the complex competition that occurs between different repair pathways. As an additional option for heavy metal exposure evaluation, here we implement two experimental approaches that provide an insight into the dynamics between repair pathways and information on the repair outcome of exogenously induced double strand breaks [21–24]. Using these complementary reporter assay systems, we determined which DSB repair mechanism predominates in mammalian cells with different concentrations of heavy metal exposures. Furthermore, our data demonstrate that heavy metal exposure skews the mechanism used by cells to repair DSBs, thereby directly affecting the outcome of the repair.

## Materials and Methods

### Chemicals

Cadmium chloride (CdCl_2_), nickel chloride (NiCl_2_), and arsenic trioxide (AsO_3_) were purchased from Fisher Scientific. Dilutions of heavy metals used in these assays were made using a filter sterilized stock solution diluted in antibiotic free DMEM medium.

### U2OS cell assay system and cell cytometry analysis

**Fig 1:**
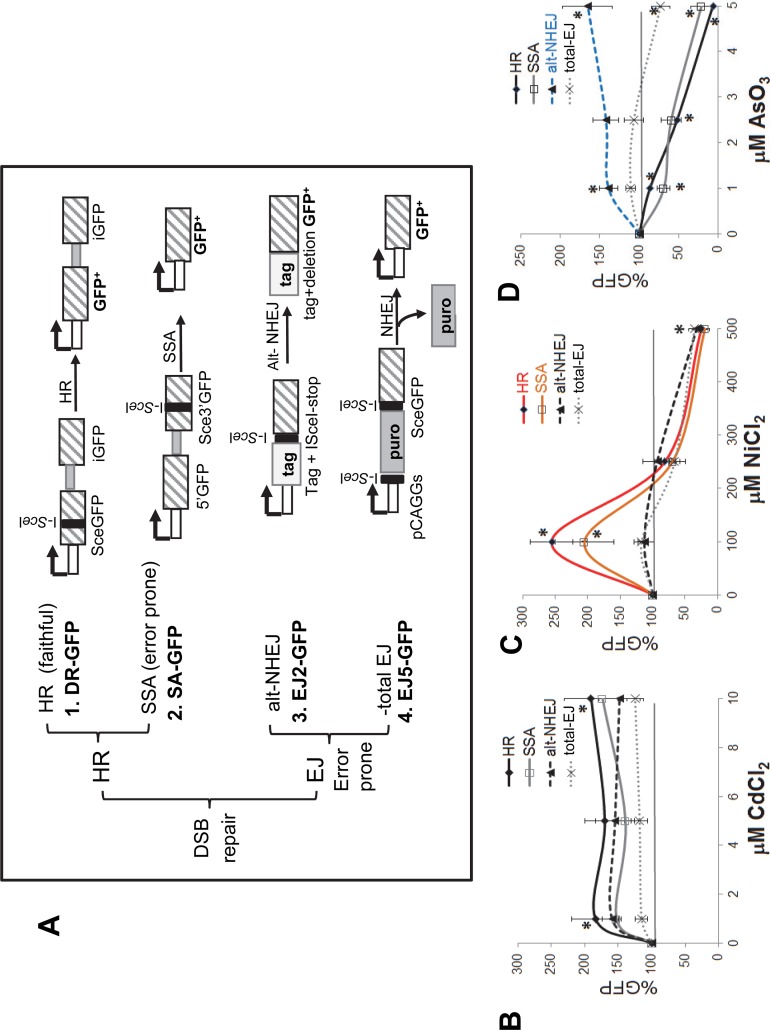
Exposure to different heavy metals alters the selection of DNA repair pathway used to repair exogenously induced double strand breaks. **A.** A schematic of the four green fluorescent protein (GFP)-based reporter cassettes are shown. These reporter cassettes are designed to provide quantitative information on the outcome of the double strand break (DSB) repair [23]. The exogenously induced DSBs are repaired by Homologous Recombination (HR) or Non-homologous End Joining (NHEJ). Depending on which pathway is utilized to repair the break the outcome will be faithful restoration of the DNA sequence or a mutated outcome (error-prone repair). The four reporter cassettes are: 1) DR-GFP for homology-directed repair (HR), 2) SA-GFP for single strand annealing (SSA) repair or non-allelic homologous repair (NAHR), 3) EJ2-GFP for alternative non-homologous end joining (alt-EJ) and 4) EJ5-GFP for total end joining (total EJ). The DR-GFP cassette contains the SceGFP cassette that is interrupted by an I-*Sce*I site (black bar) and followed downstream by a 5’and 3’ truncated fragment of GFP (iGFP). The I-*Sce*I induced DSB is repaired by HDR using the iGFP sequence as the template, which can lead to a GFP+ product. The SA-GFP cassette contains a 5’ fragment of GFP (5’GFP), and a 3’ fragment of GFP (Sce3’GFP) disrupted by an I-*Sce*I site. Repair of the DSB in Sce3’GFP by single strand annealing will generate a GFP+ product and cause a deletion (unfaithful repair). The EJ2-GFP cassette contains an expression cassette for a tagged version of an I-*Sce*I disrupted GFP and a series of stop codons, which is flanked by eight bases of homology. Repair by alternative end joining (alt-EJ) that deletes the stop codons, restores the GFP coding frame, and bridges the eight bases of flanking homology, leads to a GFP+ product. The EJ5-GFP cassette contains the pCAGGS promoter separated from the GFP coding sequence by a puromycin selection cassette (puro) flanked by I-*Sce*I sites. Shown is the outcome of a distal-EJ (total EJ) event where the puromycin sequence is removed by the joining of the two flanking sequences in a manner that leads to the expression of the GFP+ product. Only the HR pathway through homology-directed repair can restore the genomic damage faithfully. In contrast, the other repair outcomes reflect mutagenic changes that occurred during repair of the DSB. Cell lines containing the GFP-reporter systems DR-GFP for homologous recombination (HR, -♦ -), SA-GFP for single strand annealing (SSA -□-), EJ2-GFP for alternative non-homologous end joining (NHEJ, --▲--) and EJ5-GFP for non-homologous end joining (total EJ, ^…^x^…^) were treated with different amounts of: **B.** cadmium chloride, **C.** nickel chloride and **D.** arsenic trioxide and. The Y-axis shows the percent fluorescence (% GFP) normalized using the results from the I-*Sce*I transfected and untreated cells as baseline, which was set to 100%, and highlighted as a solid horizontal line across the graph. Heavy metal treatments increasing a particular repair outcome will show signals above 100% while inhibitory effects will show signals below the 100%. Asterisks indicate results significantly different from untreated cells t-test, *P* < 0.05; n ≥ 3. Contrasting results between different metal exposures are highlighted in red and blue.

The first assay consists of a previously established panel of four U2OS osteosarcoma cell lines (ATCC HTB-96) [23], each harboring a distinct chromosomal reporter cassette: 1. DR-GFP, 2. SA-GFP, 3. EJ2-GFP, and 4. EJ5-GFP (details in Fig 1A). The cells were grown in a humidified 6% CO_2_ incubator at 37°C in Dulbecco’s minimal essential medium (DMEM) supplemented with 10% fetal bovine serum. Cells were seeded in 12 well plates at a density of 1.5 x 10^5^ cells/well. The following day the cells were transfected with 1 μg of the I-*Sce*I expressing plasmid, pSCBase [21], using Lipofectamine 2000 (Life Technologies) for three hours, following the manufacturer’s protocol. Cells were additionally evaluated in parallel by cotransfecting the ptd Tomato-N1 vector (Clontech) to determine transfection efficiency and to serve as toxicity control. Following removal of the transfection cocktail, cells were grown in medium with the selected dose of the heavy metals for the duration of the experiment. Cells were harvested 68–72h post transfection by trypsinization and resuspended in phosphate buffer saline (PBS). Cells were then filtered through a mesh strainer (test tubes from Becton, Dickson and Company, BD 352235) to remove clumped cells. Flow cytometry was carried out on a Becton Dickinson LSR II flow cytometer (Louisiana Cancer Research Consortium (LCRC) FACS Core) and 50,000 events were collected.

### AARP cell assay system and rescue of repair events

**Fig 2:**
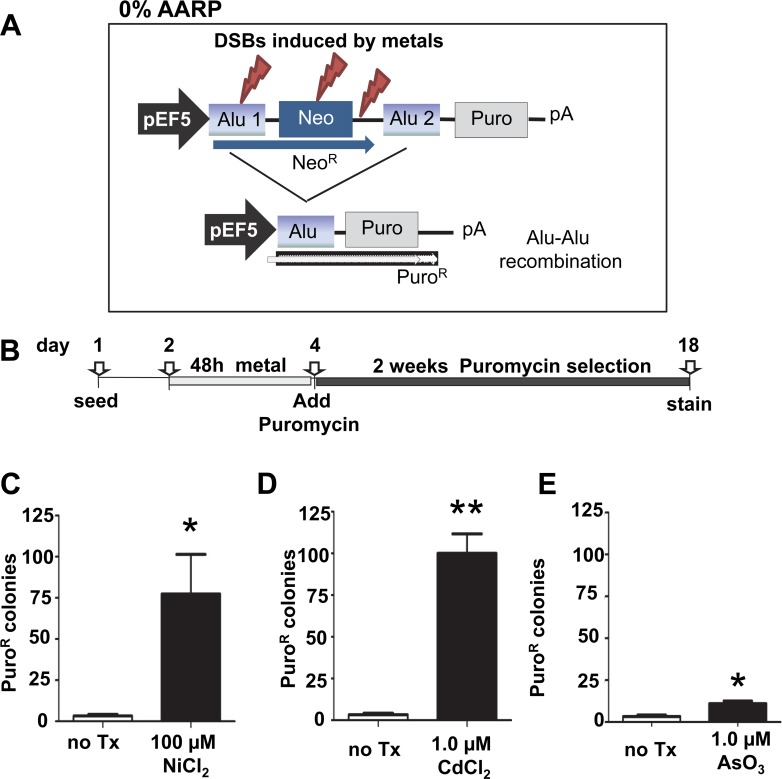
Exposure to heavy metals induces deletion events. **A.** A schematic of the 0% AARP cassette is shown. The cassette is stably integrated into an FRT site of the HEK293 cell line. The basic construct consists of a promoter upstream of two identical Alu sequences (*i*.*e*. 0% divergence between Alu1 and Alu2) separated by approximately 1.1 kb which contains an I-*Sce*I site. The vector is designed so that only Neo (blue) is expressed driven by the pEF5 promoter (gray arrow). DSBs can be exogenously induced by transfection of an I-*Sce*I expression vector or to evaluate induction of DSBs by xenobiotics like heavy metals. In this approach, only those DSBs generated by metal exposure that occur near the cassette sequences are detectable. Events that are repaired by deleting the sequence between the Alus will allow expression of Puro^R^ (light gray). All events detected by this assay are repair events that result in a mutagenic outcome. Neo = neomycin resistance gene, pA = polyadenylation signal, Puro = puromycin resistance gene, pEF5 = elongation factor 5 promoter **B.** A schematic of the protocol of the time line of the assay is shown. Cells are seeded and 16–24h later they are exposed to medium with or without the heavy metal for 48 h. Then the medium is removed and replaced puromycin medium for selection. Fourteen days post-selection, cells are stained in crystal violet solution and the puro^R^ colonies are counted. Three heavy metals were evaluated: **C.** 100 μM NiCl_2_ **D.** 1 μM CdCl_2_ and **E.** 1 μM AsO_3_. Data represent the mean with standard error of the mean from at least three independently experiments for each condition. Statistical differences are indicated as *P<0.05; **P<0.001 (two-tailed two sample Student’s T-test).

Stable cell lines containing a single copy of one of the Alu-Alu recombination-Puro (AARP) variant cassettes in the FRT site of HEK 293 cells (Life Technologies) have been previously described [24]. Different AARP cassettes containing Alu elements that either share sequence homology (0% AARP) or have sequence divergence of 5%, 10%, or 15% were used (sequences of the cassettes are provided in S6 Table). A schematic of the AARP construct is shown in Fig 2A. Three individual clones were selected from each AARP “flipped-in” stable cell line to evaluate heavy metal exposure effects. Cells were grown in DMEM + 10% FBS and 400 μg/ml G418 to select against background recombination events. The different clonal AARP_ HEK cell lines were seeded in T-75 flasks at a density of 1 x 10^6^ cells/flask. For an exogenous source of DSBs, cells were transfected 16–24h post-seeding with 1.0 μg I-*Sce*I expression plasmid pSCBase [21], using Lipofectamine and PLUS Reagents according to manufacturer's protocol (Life Technologies). After three hours, the transfection medium is replaced by either complete medium (no treatment control) or medium containing the metal to be evaluated. After a 48 hour period of exposure, the metal treatment was removed and the cells were grown for two weeks using selection medium (containing 1.3 μg/ml puromycin) to obtain resistant (puro^R^) colonies for the rate assay and PCR analyses. Cell colonies were fixed and stained for 30 minutes with crystal violet (0.2% crystal violet in 5% acetic acid and 2.5% isopropanol). A schematic for the experimental timeline is shown in the figure for each corresponding experiment.

### Rescue and sequencing of repaired events

**Fig 3:**
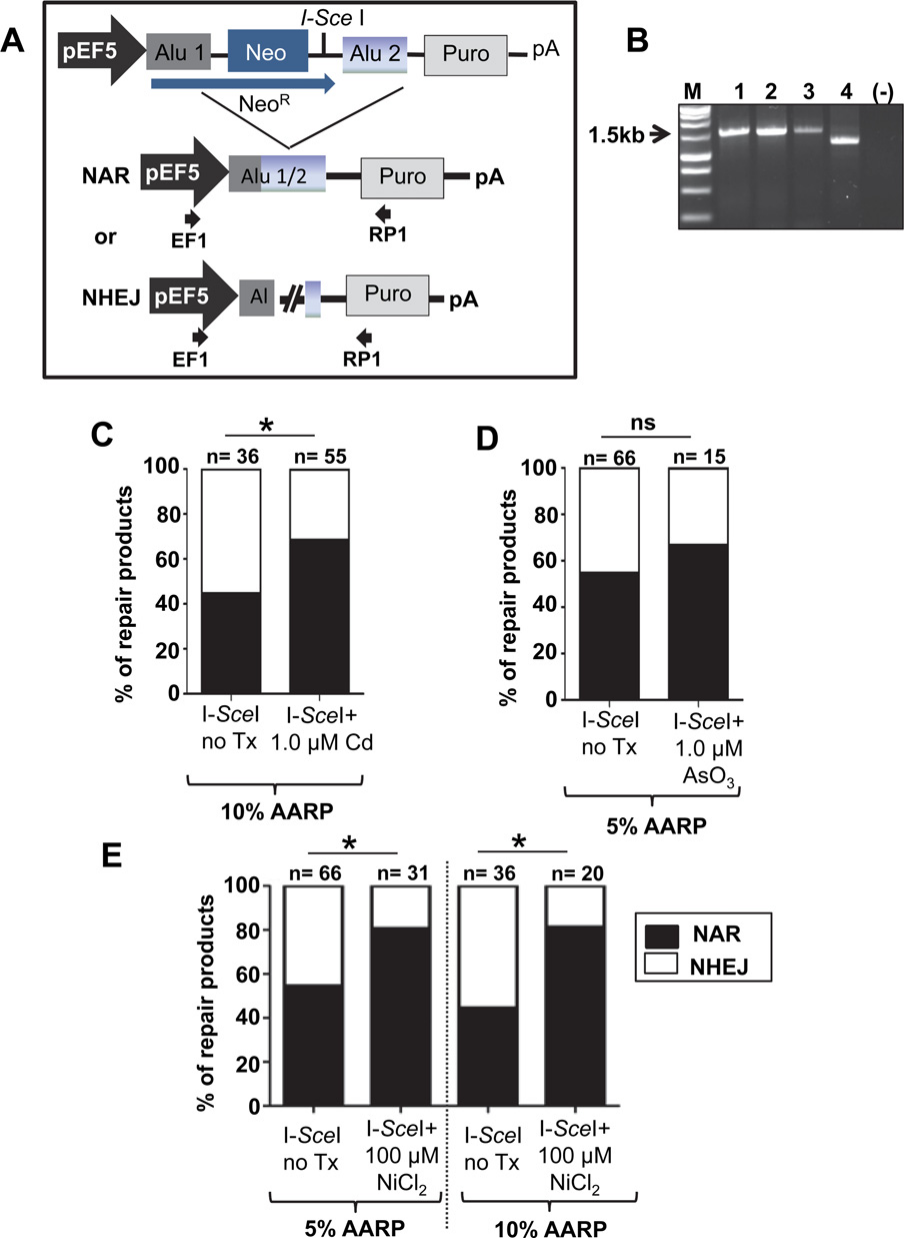
Heavy metal exposure favors NAR repair over NHEJ repair of induced DSBs. Cells containing two different AARP constructs with sequence diverged Alus (5 and 10% sequence variation between Alu1 and Alu2) were used. The cells were transfected with an I-*Sce*I expressing construct and allowed to repair in the presence or absence of heavy metals. Individual puro^R^ colonies were selected for DNA extraction. PCR products of the repaired event from DNA extracted from the individual colonies were sequenced and analyzed. The DSB repair can occur by: non allelic Alu/Alu recombination (NAR) or by Non Homologous End joining (NHEJ) driven repair through alt-NHEJ or classic NHEJ (C-NHEJ). The type of repair (NAR or NHEJ represented in **A**) is verified by sequencing PCR products of the genomic region from individual puro^R^ clones (primers F5 and R5 are shown as black arrows). An example of a PCR analysis is shown in **B**, with colonies 1–3 showing a 1.5 kb product consistent with NAR repair and colony 4 with a smaller product consistent with EJ type of repair. The Alu sequence variation allows for mapping of recombination sites in the recovered events and the events were classified as NAR (black) or EJ (white). Three heavy metals were evaluated: **C.** 1 μM CdCl_2_ **D.** 1 μM AsO_3_ and **E**. 100 μM NiCl_2_ Statistical differences are indicated as *P<0.05; (Fisher’s exact test). Numbers above each column represent the total colonies analyzed, details in Table 1.

The puromycin resistant colonies were selected and individually grown to extract DNA using the DNeasy® Blood & Tissue Kit (Qiagen) following the manufacturer’s recommended protocol. PCR amplification (38 cycles, 1min 94°C, 1min 60°C, 2min 68°C, plus a final extension 10 min 72°C) was performed using Platinum PCR SuperMix (Thermo Fisher Scientific) with primers designed to amplify the sequence located between the EF-1 promoter and the puromycin resistance gene using the following primer sets: EF1 5'-GAGAATCGGACGGGGGTAGT-3' and RP1 5'-GCTCGTAGAAGGGCAGGTTG-3'. The location of the primer annealing sites is shown as black arrows in Fig 3A. PCR amplification with these primers will make a product of 2964 bp from a cell that with an intact site (faithful repair) and a product of 1508 bp from cell that have undergone DNA repair through recombination between the two Alu elements and a variety of smaller sized products for cells that have likely undergone repair through end joining (EJ). PCR products were cloned into pGEMTeasy (Promega) or TOPO-TA (Thermo Fisher Scientific) for sequencing. The sequences were classified into two basic categories: NHEJ or NAR, which were then further subclassified into the different types of events for each category. The raw numbers and sequencing data are provided in S1–S5 Tables and alignments are shown in S7 and S9 Figs.

### Cell cycle analysis

I-*Sce*I transfected 0% AARP HEK cells were trypsinized and harvested after a 48 h incubation with the selected heavy metals. Cells were fixed for ≥ 24 hrs with ice cold 70% ethanol at -20°C. After fixation cells were washed once with PBS and incubated in PI/Triton X-00 staining solution (20 μg/ml propidium iodide, 0.1% (v/v) Triton X-100 and 0.2 mg/mL RNAse A in PBS) for at least 2 hours. Flow cytometry of the stained cells was carried out on a Becton Dickinson LSRII using DiVA Software (Louisiana Cancer Research Consortium (LCRC) FACS Core) and 50,000 events were collected. The data was analyzed using Modfit Software (Verity Software House).

## Results

### DSB DNA repair pathway evaluation: ex-vivo GFP reporter assay system

To determine the influence of heavy metal exposure on DSB repair outcomes, we utilized a previously published cellular system that provides quantitative data on the utilization frequency of different DNA repair pathways after DSB induction [22]. This system consists of four U2OS cell lines (ATCC HTB-96) [23] that contain stably integrated single copies of one of four green fluorescent protein (GFP)-based reporter cassettes. The four cell lines are: 1) DR-GFP for homology-directed repair (HR), 2) SA-GFP for single strand annealing (SSA) repair, 3) EJ5-GFP for total end joining [total EJ: combination of Classic-NHEJ (C-NHEJ) and alt-NHEJ] between distal ends of two DSBs (Distal-EJ) and 4) EJ2-GFP for alternative non-homologous end joining (alt-NHEJ). The targeted double strand break is generated by transfecting a plasmid that expresses the rare-cutting endonuclease I-*Sce*I that recognizes an 18 bp sequence within the reporter cassette. The use of the I-*Sce*I endonuclease provides a constant and controlled source of DSBs that occur in a specific site, limiting experimental variability. In this context, the terms “HR, SSA, total EJ and alt-NHEJ” are used to describe a defined repair outcome and the mechanism of repair. Details of the reporter cassettes are shown in Fig 1A. Of the four outcomes evaluated, SSA, total EJ and alt-NHEJ recombination cassettes measure the amount of DSBs repaired through processes that can introduce sequence changes, contributing to unfaithful repair. Each reporter cassette provides quantitative information on the individual repair pathway evaluated. Thus, direct comparisons between exposed cells and unexposed cells can be performed.

### The effect of heavy metal exposure on double strand break repair varies between metals and exposure dose

The U2OS reporter cell lines were transfected with an I-*Sce*I expression plasmid or a red fluorescent protein (RFP) expression plasmid (used as toxicity control). Immediately after transfection the cells were incubated with medium containing varying amounts of cadmium chloride (0–10 μM), nickel chloride (0–500 μM), or arsenic trioxide (0–5 μM) for 72 hours. The cells were harvested and the number of GFP positive cells (repair signal) or RFP positive cells (transfection and toxicity control) out of a total of 50,000 was determined by cell cytometry. An example of a representative FACS result is shown in S1 Fig. The GFP signals were normalized to the untreated control, which was arbitrarily set at 100%. The untreated control represents the mean number of cells repaired by the DNA repair pathway analyzed after transfection with *I-SceI*. In our experimental setting, heavy metal exposure can have three outcomes: 1- the number of cells repaired by the pathway analyzed remains the same as untreated control; 2- a reduction in the number of cells repair by the pathway analyzed, suggesting a potential inhibitory effect of the metal on the pathway; or 3- an increase in the number of cells repaired by the pathway analyzed, suggesting that this pathway may have taken a compensatory role for the inhibition of another pathway. Note that an increase in GFP signal does not necessarily represent an absolute upregulation of the DNA repair pathway by the metal. Instead, it may represent a decrease in a different pathway, allowing the DNA repair pathway being tested to compete more effectively.

The heavy metals tested in these experiments are capable of generating DNA breaks in a neutral comet assay (S2 Fig). However, in this assay system the doses of heavy metals evaluated were unable to induce a signal above background (S3 Fig). This result is not surprising; as the DSBs induced by the heavy metal exposure (*e*.*g*. nickel and cadmium) would occur randomly throughout the genome and the likelihood that a DSB occurring within the cassette is very low. However, this assay is useful to evaluate the effects of heavy metal exposure on the specific forms of DSB repair, by introducing an exogenously induced DSB by using an I-*Sce*I expression vector to cut the cassette at the ideal location. The combined results of analyses of all four DNA repair pathways provides an insight into how each heavy metal exposure affects the outcome of the DSB repair. Each heavy metal exposure presented unique overall effects on the different DNA repair pathways (Fig 1B–1D), which are as follows:

#### Cadmium

All three doses of cadmium chloride tested (1, 5 and 10 μM) showed no significant increase in the number of GFP+ cells for most types of the DSB repair pathways evaluated (Fig 1B). Some doses showed a modest increase in the DR-GFP+ cells suggestive of a potential increase using repair by homologous recombination (HR).

#### Nickel

Nickel chloride was the only heavy metal tested that showed a biphasic response where the signal observed dramatically varied depending on the dose. Exposure to 100 μM NiCl_2_ increased the DR-GFP+ and SA-GFP+ cells between 2–2.5 fold, indicating a preferential increase in the use of repair by HR and single strand annealing (SSA) (Fig 1C, paired t-test *P<0.05). However, this increase may not represent the maximal stimulation as lower doses were not evaluated. In contrast, the highest dose tested (500 μM) significantly inhibited the GFP signal in all cells.

#### Arsenic

All three doses of arsenic trioxide (1, 2.5 and 5 μM) favored resolution of DSBs by alt-NHEJ (Fig 1D) and significantly inhibited both HR and SSA (Fig 2C, paired t-test *P< 0.01). We observed a dose response, where the number of the alt-NHEJ GFP+ cells increased with the higher concentrations of arsenic. The highest dose tested (5 μM AsO_3_) showed a slight inhibition of the total EJ signal (paired t-test *P< 0.001) and significantly inhibited both HR and SSA (Fig 1C, paired t-test *P*<0.01).

These results show that each heavy metal had unique effects on the DSB repair outcome showing varied results depending on both the specific metal tested and the dose. Several of the doses tested favored error-prone repair further corroborating heavy metals as potential co-carcinogenic agents [25].

### The Alu-Alu recombination reporter assay system

Alu elements are primate-specific SINEs (Short, INterspersed Elements) that have amplified over the past 65 million years comprising approximately 11% of the human genome with well over one million copies [26]. DSBs in and near repetitive sequences, such as Alu elements, are a potent source of genomic instability. Alu elements have been shown to serve as substrates for non-allelic recombination (NAR) through SSA [27–30], as well as participating on NHEJ events [24,31–33]. We used our previously published Alu-Alu Recombination assay system [24], to further investigate the influence of repeated sequences on DNA repair outcomes after heavy metal exposure. We first utilized the AARP cassette that contains Alu elements with identical sequences [0%; *i*.*e*. non-diverged (see Fig 2A and 2B for a schematic of the cassette and outline of the assay timeline)]. The AARP cassette is inserted into the FRT-site of Human Embryonic Kidney cells (HEK 293 cell line), providing the ability to change reporter cassettes and maintain constant the genomic location for all experiments. The assay allows for detection of NAR recombination events and a subset of non homologous end joining types of repair (NHEJ and alt-NHEJ). Note that this assay system does not detect repair by HR (gene conversion) or small canonical NHEJ events as their repair outcome would not introduce sufficient changes to the cassette to allow the expression of the puromycin resistance gene. However, it provides data on the two most mutagenic forms of repair that cause genomic deletions, which is the main focus of this manuscript.

### Exposure to heavy metals favors Alu-Alu mediated recombination

Because heavy metals can cause DSBs by oxidative stress, we evaluated if the exposure would promote Alu-Alu mediated recombination in our assay system. Three independent FRT-integrated cell clones of the 0% AARP HEK were selected and incubated for 48 h with 1.0 μM CdCl_2_, 100 μM NiCl_2_ or 1 μM AsO_3_. Cells grown in the growth medium (*i*.*e*. no treatment) were used as a negative control. After the metal treatment, cells were grown for two weeks under puromycin selection (to measure recombination) or neomycin selection (to evaluate potential toxicity of the metal treatment on cell growth). There was no evidence of toxic effects observed under these exposure conditions (S4 Fig). Treatment with nickel, cadmium and arsenic induced a significant number of puromycin colonies (Fig 2C–2E, paired t-test *P<0.05; **P<0.001). Although significant, arsenic trioxide only showed a very modest induction of puro^R^ colonies (Fig 2E). These results demonstrate that a 48h exposure to these doses of heavy metals were mutagenic and sufficient to promote the loss of genomic sequence to allow expression of the puromycin resistance gene. Recovery and sequencing of some representative colonies confirmed that Alu-Alu mediated NAR had occurred. However, we are unable to distinguish if the results stem from the generation of DSBs from collapsed replication forks [34] or effects on DNA repair due to the heavy metal exposure.

### Exposure to cadmium, nickel and arsenic favors repair of DSBs through non-allelic recombination (NAR)

For the following analyses, we used a different set of stable HEK cell lines that carry one of the AARP cassette variants that contain non-identical Alu elements [24] (Fig 3A) and are more representative of the typical variation between Alu elements present in the human genome. Three AARP variants containing Alu elements with 5%, 10% or 15% sequence divergence between Alu1 and Alu2 were tested. As previously published, the overall number of puro^R^ colonies observed declined when evaluating metal treatments using the diverged AARP cassettes [24] and some heavy metals showed no effect. For example, a 48 h exposure of 100 μM NiCl_2_ only induced a very modest signal when using the 5% and 15% AARP containing cells (S5 Fig).

To further explore if the heavy metal exposure affected repair of DSBs, we transfected the cells with an I-*Sce*I expression vector to provide a source of exogenous DSBs that will specifically cleave between the two Alu sequences (see schematic Fig 3A). With this approach, we are now able to evaluate the effects of heavy metal treatment specifically on DSB repair and in the presence of imperfect Alu homeologies. The heavy metal treated I-*Sce*I transfected cells showed no significant increase in puro^R^ colonies compared to the untreated control (S6 Fig). Isolated DNA from individual puro^R^ colonies was PCR amplified and sequenced to analyze the repaired events (Fig 3A and 3B, S1–S6 Tables). Although the number of puro^R^ colonies was not significantly different, recovery and sequencing of multiple individual repaired events demonstrated the DSB repair outcomes significantly differed between the treated and untreated cells (Table 1). We observed that the relative proportion of NAR to NHEJ repaired events changed in the treated cells. All three heavy metal treatments favored Alu-mediated NAR over NHEJ type of events when compared to the no treatment control (Fig 3C–3E). This result may represent either an increase in NAR repair or a decrease in NHEJ or a combination of both. Because these heavy metals have been previously shown to be inhibitory to many repair proteins we speculated that in this scenario it is likely NHEJ is decreased. However, further studies are need for confirmation.

**Table 1:** Number of recovered events.

**Condition**	**NAR (%)**	**NHEJ (%)**	**Total**
10% AARP *I-SceI* no Tx	16 (44%)	20 (56%)	36
10% AARP *I-SceI* 1.0 μM CdCl_2_^a^	38 (69%)	17 (31%)	55
10% AARP *I-SceI* 100 μM NiCl_2_^b^	17 (85%)	3 (15%)	20
5% AARP *I-SceI* no Tx	32 (48%)	34 (52%)	66
5% AARP *I-SceI* 1.0 μM ASO_3_^c^	10 (67%)	5 (33%)	15
5% AARP *I-SceI* 100 μM NiCl_2_^d^	25 (81%)	6 (19%	31

Statistical significance calculated by Fisher’s exact test between treatment and control

^a^ p = 0.0287

^b^ p = 0.0043

^c^ p = 0.3919

^d^ p = 0.0074

**Fig 4:**
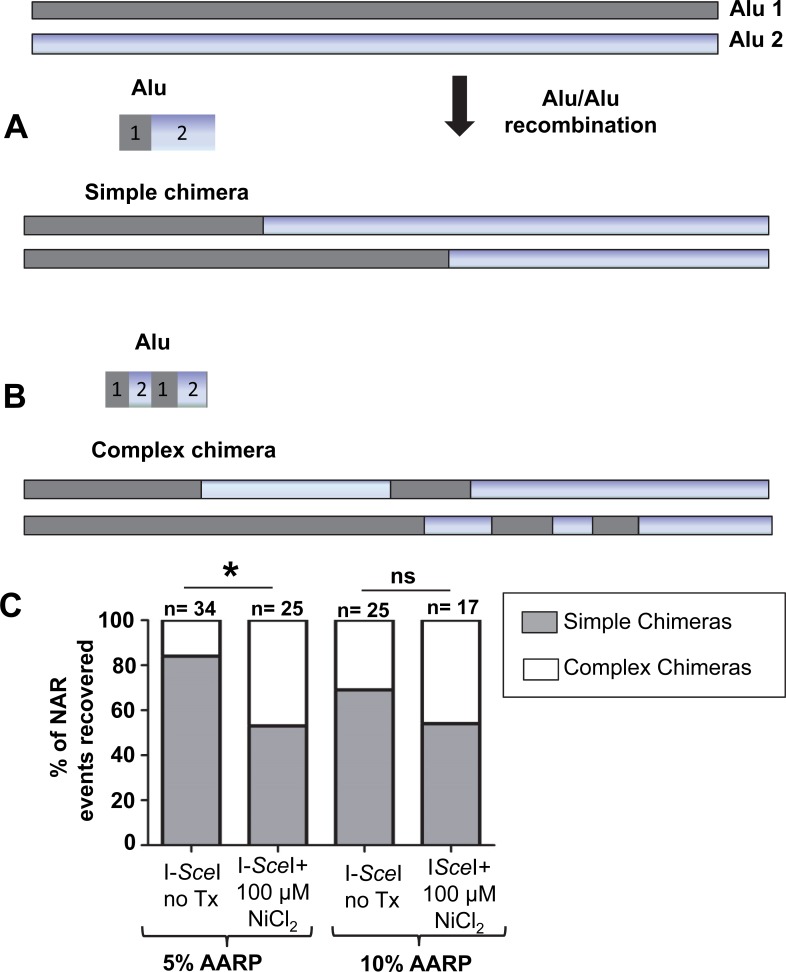
Distribution of single and complex chimeras. Alu-Alu recombination can generate two types of outcomes: simple chimera or complex chimera. **A.** A simple chimera shows only one recombination site (left portion of the Alu sequence chimera derived from Alu1 and right portion derived from Alu2). **B.** A complex chimera shows multiple recombination sites with intermixed regions of the chimera derived from the two Alu sequences. Schematic of actual recovered outcomes are shown below the diagrams. **C.** The 5 and 15%AARP HEK 293 cell line was incubated with media containing 100 μM NiCl_2_. Untreated cells were used as the reference control. Sequence analyses of the recovered events showed that two types of Alu-Alu recombination products can be observed: simple and complex. NAR events recovered from the nickel treated cells were further classified as single (gray) or complex chimeras (white). The results are expressed as mean ± SD of three independent experiments. Statistical differences are indicated as *P<0.05; (Fisher’s exact test); ns = not significant. Numbers above each column represent the total colonies analyzed; sequence alignments are shown in S7 Fig.

There are two basic types of Alu-mediated NAR outcomes that can be observed: simple chimera or complex chimera. A schematic of both types of outcomes is shown in Fig 4. We previously showed that complex chimera arise from the RAD52-dependent annealing of the strands from the two diverged Alu elements that have escaped heteroduplex rejection and seem likely to be due to SSA [24], while the simple chimera are likely to be the result of alt-NHEJ where the microhomologies are ‘in-register’ within the Alu elements [35]. Such an annealed SSA-like product would be a substrate for mismatch repair processes, which is suggested to produce an Alu element containing patches from each Alu element *i*.*e*. a complex chimera [36]. Sequence evaluation of the Alu-mediated NAR events demonstrated that in our system, nickel exposure favored the generation of complex chimera as compared to the untreated control (*P<0.05; Fisher’s exact test; Fig 4 and S2–S5 Tables and S7 Fig).

The choice of DNA repair pathway depends on both the type of DNA lesion and the cell-cycle phase. Thus, we investigated the effect of our exposure conditions on the cell cycle, as some heavy metals are able to alter the distribution of cells in the different cell cycle phases [37–39]. Using the same experimental conditions, the I-*Sce*I transfected AARP HEK cells were exposed to 1 μM CdCl_2_, 1 μM AsO_3_ and 100 μM NiCl_2_ for 48h. After incubation with the heavy metal, the cells were harvested and evaluated using flow cytometry. No significant difference was observed between treated and untreated cells (S8 Fig). This suggests that the effects observed on DNA repair outcomes resulting from the exposure conditions used in this study are unlikely due to effects on the cell cycle of HEK cells.

### Exposure to cadmium, nickel and arsenic alters the sequence characteristics of EJ DSB repair outcomes

**Fig 5:**
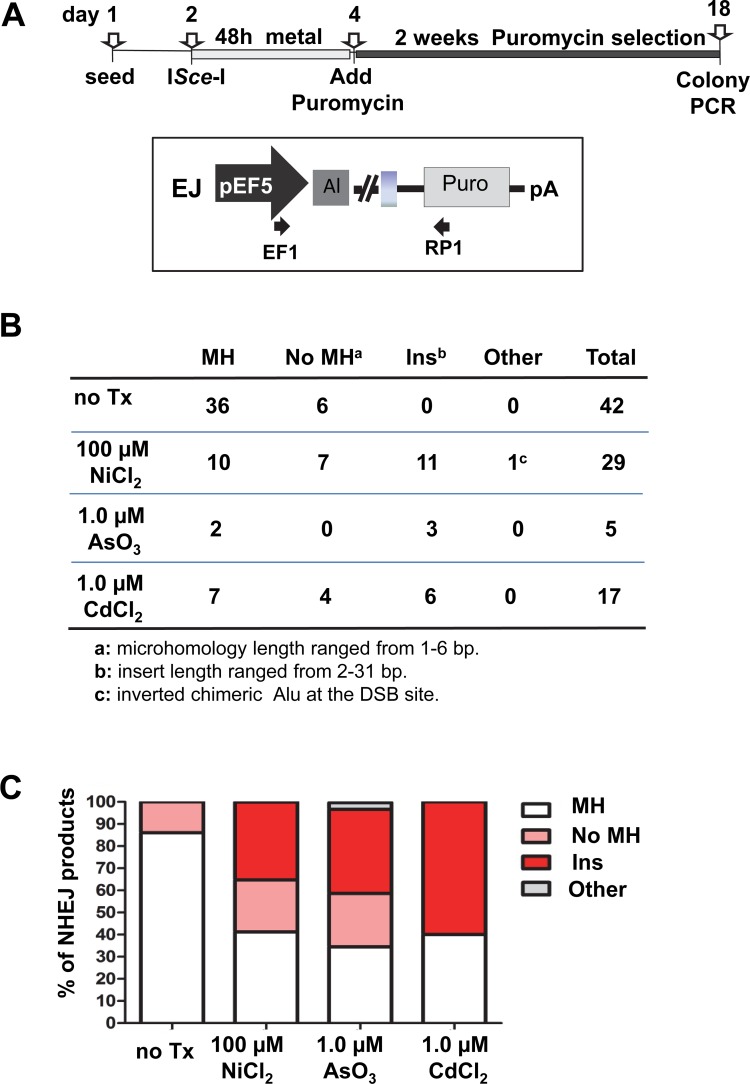
Heavy metal exposure alters the sequence characteristics of EJ DSB repair outcomes. **A.** A schematic of the protocol of the time line of the assay is shown. **B.** Table showing the number of recovered events classified as end-joining products of I-*Sce*I induced breaks of cells exposed to: no treatment control, 1 μM CdCl_2,_ 100 μM NiCl_2_, and 1 μM AsO_3_. Events rescued from all experimental conditions were pooled and classified into events showing microhomology (MH), no microhomology (noMH) and insertions (Ins) at the repair site. Note that no insertions were ever observed in the EJ events from unexposed cells. **C.** Stacked column chart shows the relative proportion of the types of end-joining products rescued from repaired I-*Sce*I induced breaks of cells exposed to 100 μM NiCl_2_, 1 μM CdCl_2_ and 1 μM AsO_3_. “Other” represents one event showing the presence of one inverted chimeric Alu sequence at the DSB site. Sequence data obtained from untreated cells were used as the no treatment control.

Although the proportion of DSBs repaired by EJ events relative to NAR events was reduced with the heavy metal treatments (Fig 3C–3E), the breakpoint junctions of the EJ repair events also showed different sequence characteristics when compared to the untreated control (Fig 5). The vast majority of sequenced breakpoint junctions from untreated cells with EJ repair events showed microhomology (MH). In contrast, all heavy metal treatments showed a reduction in the number of events with microhomology and the appearance of events with non-templated base insertions (Ins) at the repair site (Fig 5C and S9 Fig and S5 Table). This is notably different, as no insertions were observed in the untreated control. One nickel treated event showed a recombination that yielded a chimeric Alu in an inverted orientation that was classified as “other”.

## Discussion

Cells have multiple DNA repair pathways that compete with one another to repair DSBs. The DSB repair pathways can be divided into two basic groups: repair that uses some form of repair that uses sequence homology (HR and SSA) and repair that involves non-homologous end joining (NHEJ) [1,2]. Our experimental data clearly indicate that heavy metal exposure significantly influences DSB DNA repair outcomes. Our observations indirectly support the current literature demonstrating that heavy metals can inhibit individual proteins from multiple repair pathways based on specific functional requirements of affected proteins. We propose that our results are a consequence of the ability of heavy metals to affect or inhibit the function of critical repair proteins influencing the choice of DNA repair pathway and therefore changing the final outcome of the repaired DNA. For example, we observe that arsenic favors resolution of DSBs through alt-NHEJ (Fig 1D). A previous report showed that arsenic trioxide inactivates Rad51 (HR) through the suppression of AKT activity [40]. Thus, our results may reflect the inability of the cell to recruit Rad51 in the presence of arsenic, allowing microhomology-mediated annealing and promoting DSB repair by alt-NHEJ. Furthermore, both cadmium and nickel seem to favor resolution of DSBs by HR and SSA in addition to increasing Alu-mediated deletions (likely through SSA). We speculate that these repair pathways are favored because the heavy metals interfere with C-NHEJ by inhibiting binding of the Ku complex to the exposed DNA ends to prevent resection. However, further studies are needed at this time to validate this. Strikingly, we also observed an increase in the number of sequence insertions at the repair sites of DSBs repaired by alt-NHEJ in cells treated with nickel, arsenic, or cadmium when compared to non-treated cells (Fig 5). Our study shows for the first time that heavy metal exposure increases sequence insertions at DBS repair sites. In this instance, the observed insertions could be a reflection of the metals interfering with the enzymatic processes of alt-NHEJ repair proteins.

**Fig 6:**
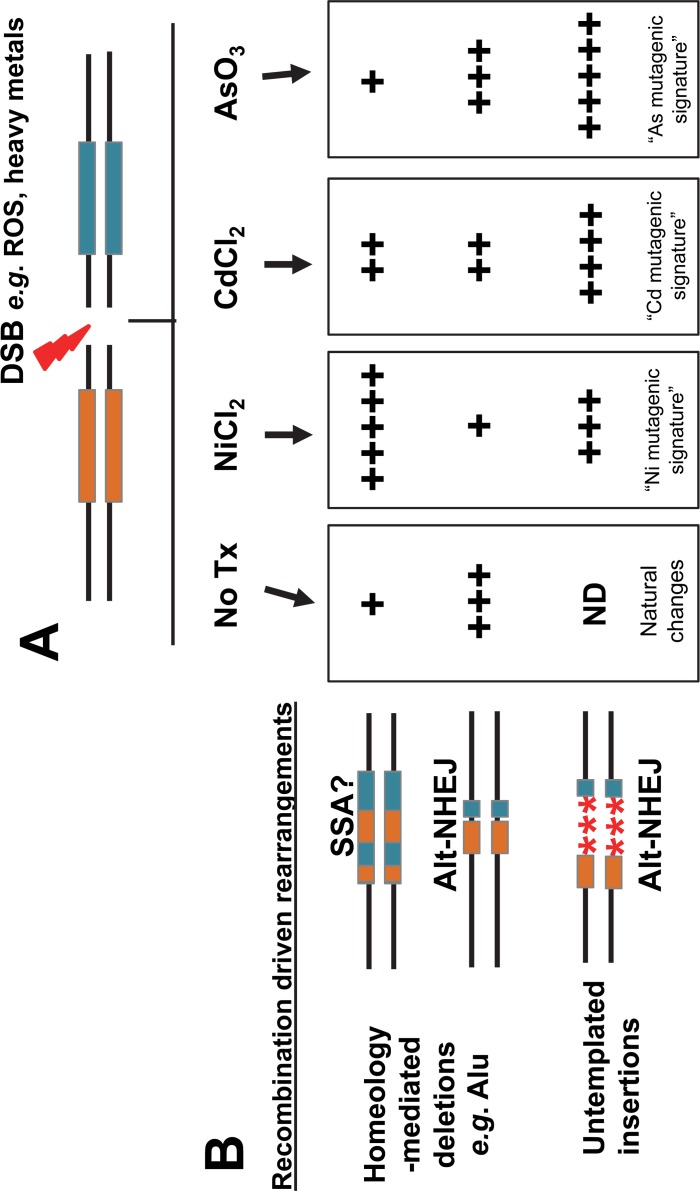
Role of heavy metals as promotors of genetic instability through the accumulation of genetic damage by favoring error-prone DNA repair. **A.** DSBs are consistently occurring in cells either as a byproduct of natural processes or due to external insults such as heavy metal exposure. DSBs near repetitive sequences, such as Alu elements (shown as orange and blue boxes) will result in different repair outcomes usually with one type being favored. **B.** When compared to an untreated control (no Tx), our data show that exposure to different heavy metals can differentially alter the choice between pathways to promote error-prone repair by favoring spontaneous recombination between repeat sequences (*e*.*g*.Alu); homeology-mediated deletions which occur through the NAR or Alt-NHEJ repair; and untemplated insertions during repair by alt-NHEJ. The proportion of the different types of repair outcomes varies between the different heavy metal treatments. The overall effect of heavy metal exposure will favor the accumulation of specific genomic changes and loss of genomic sequence contributing to signature mutagenic changes as a manifestation of the exposure. ND = not detected.

By using these assay systems, we demonstrated that the DSB repair outcomes vary between distinct heavy metal exposures causing the accumulation of a different set of mutagenic changes in the genome. The potential power in these data comes from the ability to generate a knowledge base that will allow prediction of exposure-associated genomic signatures of damage. Not only will the data catalogue the type of genetic damage heavy metals may induce, but will also provide insight into the mechanism behind the mutagenic changes. For example, cells exposed to a low dose of NiCl_2_ (100 μM) favored resolution of the DSBs by HR and SSA (Fig 1C) and significantly favored NAR repair of DSBs in our Alu-Alu recombination assay (Fig 3E). In addition to increasing Alu-mediated recombination events, nickel also favored the creation of complex chimeras (Fig 4), which we speculate are likely SSA repair events (Rad52 dependent). Furthermore, nickel caused a shift in the outcomes of alt-NHEJ repair with a significant increase of non-templated sequence insertions at the DSB repair site. Based on these data, we propose a scenario where exposure to non-cytotoxic concentrations of nickel will contribute to the accumulation of cells containing signature mutagenic changes at the DSB repair site (Fig 6). While the number of DSBs may not be altered, the repair itself is less precise. Therefore, we predict that nickel, cadmium and arsenic exposure may contribute to an increased accumulation of: 1) spontaneous recombination between repeated sequences (*e*.*g*. Alu) promoted by metal induced DSBs, 2) homeology-mediated deletions (*e*.*g*. NAR and alt-NHEJ), and 3) untemplated insertions at DSB sites that occurred during repair by alt-NHEJ. Each heavy metal will likely have a recognizable mutagenic signature consisting of the accumulation of the particular repair outcomes favored by the exposure. One previous report in the literature supports this model. Data from a whole genome analysis of an arsenic-related small cell lung tumor from a chronically exposed never-smoker patient identified a distinct mutational signature, which differed from general lung tumors [41]. This report was the first to demonstrate differences between a heavy metal induced tumor and other tumors at the genetic level.

Finally, we examined the role of heavy metal exposure on influencing Alu-mediated rearrangements. We specifically selected Alu because these repetitive elements densely populate the human genome, and are therefore one of the most common sources of NAR. Our data showed that nickel and cadmium exposures favored Alu-mediated NAR resulting in the deletion of the inter-Alu sequence (Fig 3). This can be of importance, as Alu sequences are enriched in GC-rich (*i*.*e*. genic) regions of the genome [26] and a significant amount of exonic sequence may be lost in cells exposed to these heavy metals. Previous studies demonstrate that some genes rich in Alu sequences are predisposed to large mutagenic rearrangements. In germline cells Alu/Alu recombination has been implicated in the etiology of numerous inherited diseases, including some cancers [42,43]. In somatic cells, recombination between Alu elements has been well documented, with the most intensively studied example involving the MLL gene [44]. Another example includes the SLC12A3 gene (Gitelman Syndrome), where 6% of the reported cases were caused by the presence of the abundant Alu sequences [45]. Furthermore, in the autosomal-dominant disease spastic paraplegia 4, 70% of the mutations in the SPG4 gene are thought to be mediated by an Alu-based mechanism. Interestingly, genetic damage due to Alu-mediated mutations is not limited to NAR. The data indicate that Alus are frequently found in break points [46,47] suggesting that Alu sequences may be prone to participate in NHEJ repair events. This type of breakpoint junctions (between an Alu element and a non-Alu sequence) was also observed in our recovered NHEJ events. It is important to note here that Alu-mediated deletions/mutations can potentially be one of the intrinsic contributors to promote metal-induced carcinogenesis.

Overall, our studies provide important information on the potential effects of heavy metals on the pathways protecting genomic integrity. Heavy metal exposure can be extremely detrimental to human health with different pathologies depending on length of exposure. Acute effects, particularly from high dose exposures, may cause significant cellular toxicity, *i*.*e*. death, leading to tissue loss and pathological damage to target organs. In contrast, chronic exposure of a low dose may not induce the loss of damaged cells but instead may allow the survival of cells with compromised genetic integrity. The effect could be compounded if the heavy metal also promotes gene expression changes and/or cell proliferation by either the activation of proto-oncogenes or further interfering with tumor suppressor genes that could favor the growth of the cells with the mutated genome. In this scenario, exposure to low doses of heavy metals may be more harmful than higher doses that induce cell death and thus eliminate potential sources of transformed cells. Because the genetic damage due to unfaithful DSB repair is irreversible with deleterious consequences on genome stability, determining the impact of heavy metal exposure on the outcome of these mutagenic events is fundamental to understand the extent of the toxicant’s pathogenesis on human health.

## Conclusion

Carcinogenic metals are ubiquitous elements, and humans are exposed to these toxicants via air, drinking water, occupational settings, and consumer products. A great number of epidemiological studies have established the carcinogenicity of nickel, cadmium, and arsenic by associating metal exposure with human cancer incidence. Our study provides evidence that arsenic, nickel and cadmium can promote mutagenic changes by influencing how the cell repairs DSBs. The influence of nickel and cadmium on DNA repair pathways may have an important role in metal-associated carcinogenesis at concentrations lower than those previously estimated. Based on the evidence shown in this study, low doses of nickel and cadmium should be a major concern for human health. While nickel and cadmium's ability to inhibit individual DNA repair proteins from specific pathways has been well documented in the literature, no other data are available regarding these metals tendency to promote mutagenic recombination. Although there are multiple proposed mechanisms of metal-induced carcinogenesis, we propose that metal-induced Alu/Alu recombination may also contribute to this process. Research aiming to further investigate the ability of heavy metals to promote mutagenic non-allelic recombination is needed in order to fully characterize their mutagenic effects.

## Supporting Information

S1 Fig Representative FACS profiles of the U2OS DR-GFP cell line. Shown is an example of the results from one experimental replicate. A logarithmic plot of the gated cells is shown using FITC-A (green fluorescence, x-axis) and PE-A (area of the pulse width, red fluorescence y-axis). Autofluorescence is detected on the diagonal, where cells showing increased green fluorescence are to the right of the diagonal. Autofluorescence are gated to determine the percentage of GFP+ cells (blue). In this example the cell line U2Os with the DR-GFP cassette was used to quantify homology-directed repair (HR). The examples shown are: **a**. untransfected untreated control to correct for background; **b**. I-*Sce*I transfected no treatment control (used as the baseline or “0%”); **c-f**. I-*Sce*I transfected with different heavy metal treatments (100–500 μM NiCl_2_ and 1 μM CdCl_2_). Note that 100 μM NiCl_2_ increases the signal relative to the control (**b**), while 500 μM NiCl_2_ decreases the signal.
(PDF)

S2 Fig Cadmium and nickel treatments induce DNA breaks. Neutral comet assay was performed for the detection of DNA breaks of cells after a 24 h heavy metal exposure. Gamma-irradiated cells were used as a positive control. Significant differences were observed for all treatments relative to the no treatment (no Tx) control [* *P* < 0.05** *P* < 0.001, Student’s paired T-test].
(PDF)

S3 Fig The metal doses evaluated did not induce signals in the U2Os DNA repair cell line assay. Shown are the examples for nickel and cadmium exposure on the four U2OS cell lines. A logarithmic plot of the gated cells is shown using FITC-A (green fluorescence, x-axis) and PE-A (area of the pulse width, red fluorescence y-axis). Autofluorescence is detected on the diagonal, where cells showing increased green fluorescence are to the right of the diagonal. Autofluorescence are gated to determine the percentage of GFP+ cells (blue). In this example the U2Os cell lines with the four different GFP cassettes were evaluated. The examples shown are: **a**. untransfected untreated control to correct for background; **b**. I-*Sce*I transfected no treatment control; **c-**.treatment with 100 μM NiCl_2_ and **d**. treatment with 1 μM. Exposure to the metals alone is not sufficient to induce a GFP signal above the background in any of the four different cells lines.
(PDF)

S4 Fig The heavy metal treatments evaluated have no adverse effect on cell growth and ability to form cell colonies under selection media. To evaluate toxicity the 0%AARP HEK cells were transfected with a plasmid expressing neomycin resistance (pIRES-EGFP, Life Technologies) and grown in the presence of 1 μM CdCl_2_, 1 μM AsO_3_,100 μM or NiCl_2_ for 48 h. The metal containing medium was removed and cells were grown for two weeks under G418 selection. Cell colonies were fixed and stained for 30 minutes with crystal violet (0.2% crystal violet in 5% acetic acid and 2.5% isopropanol) and counted. No significant decrease in colony numbers (*P*>0.05) was observed between untreated cells and heavy metal exposed cells.
(PDF)

S5 Fig Sequence divergence of the Alu elements yields lower signals on the Alu-Alu recombination system with a reduction in the total number of puro^R^ colonies. **A.** A schematic of the basic diverged AARP cassette is shown. The construct consists of a promoter upstream of two different Alu sequences (*e*.*g*. 5% divergence between Alu1 and Alu2) separated by approximately 1.1 kb. Metal induced double strand breaks (DSBs) will occur in the cassette and throughout the genome. Repaired events deleting genomic DNA that brings the EF5 promoter upstream of the puromycin gene will confer puromycin resistance. **B.** Sequence divergence of the Alu elements yields lower signals on the Alu-Alu recombination system with a reduction in the total number of puro^R^ colonies. The 5 and 15%AARP HEK cell line was incubated with media containing 100 μM NiCl_2_. Untreated cells were used as the reference control (no Tx). The results were expressed as mean ± SD of three biological replicates. (**P<0.001, Student’s paired t-test).
(PDF)

S6 Fig Metal exposure does not increase the rate of Alu-Alu recombination signal in I-*Sce*I transfected cells. The 5 and 10%AARP HEK cell lines were transfected with an I-*Sce*I expression vector and incubated with media containing either **A.** 100 μM, NiCl_2_ or **B.** 1 μM CdCl_2_for 48 h before undergoing selection for 2 weeks with puromycin. Untreated cells were used as the reference control (no Tx). The results were expressed as mean ± SD of three biological replicates.
(PDF)

S7 Fig Sequence alignment of recovered of simple and complex chimera events recovered after nickel treatment. The specific nucleotides used to identify the Alu1-Alu2 boundaries are indicated in red for Alu2 and in blue for Alu1. **A**. 5% simple chimera, **B.** 5% complex chimera, **C.** 10% simple chimera and **D.** 10% complex chimera.
(PDF)

S8 Fig The metal treatments evaluated have minimal to no effect on cell cycle. I*Sce*-I transfected 0%AARP HEK cells were incubated with media containing 1 μM CdCl_2_, 100 μM NiCl_2_, or 1 μM AsO_3_ following the same protocol used to generate the experimental data (Figs 3–5). Untreated cells were used as the reference control (no Tx). The cells were harvested after 48 h exposure time and analyzed with propidium iodide staining to assess cell cycle distribution by FACS analysis. Shown is an example of the results in **A** the raw histograms (note difference in scales) and in **B** the graph of the percentages of cells in each phase of the cell.
(PDF)

S9 Fig Sequence alignments of the break site flanking sequences of the different type of events repaired through non-homologous end joining after metal treatments. The different types of NHEJ repair events were classified as having no microhomology (no MH), having insertions, having microhomology (MH) or other (not shown). The alignments show the flanking region of the break site with are different features highlighted: Insertions (blue) and MH (yellow). The deleted region is represented by the light blue sequences within the brackets. The size of the deletion is shown on the right in red.
(PDF)

S1 Table Number of individual events recovered and sequenced (data used in Figs 3 and 4). (XLSX)

S2 Table Sequencing data from rescues Fig 3C [1.0 μM CdCl_2_ treatment]. (XLSX)

S3 Table Sequencing data from rescues Fig 3D [1.0 μM AsO_3_ treatment]. (XLSX)

S4 Table Sequencing data from rescues Figs 3E and 4 [100 μM NiCl_2_ treatment]. (XLSX)

S5 Table Sequencing data from rescues Fig 5B and 5C [1.0 μM CdCl_2_, 1.0 μM AsO_3_, 100 μM NiCl_2_ treatment]. (XLSX)

S6 Table AARP cassette sequences. (XLSX)

## Abbreviations

AARP: Alu/Alu Recombination Puromycin
alt-NHEJ: Alternative-Non-Homologous End Joining
DSBs: Double Strand Breaks
FACS: Fluorescence-Activated Cell Sorting, Green Fluorescent Protein
HR: Homologous Recombination
neo: neomycin
NHEJ: Non-Homologous End Joining
NAR: Non-Allelic Recombination
PCR: polymerase chain reaction
puro: puromycin
SINE: Short Interspersed Element (SINE)
