# Supplementary material for: Heavy Metal Exposure Influences Double Strand Break DNA Repair Outcomes
Source: PLoS One. 2016 Mar 11;11(3):e0151367. doi: 10.1371/journal.pone.0151367 (PMC4788447; doi:10.1371/journal.pone.0151367)
Supplement: S1 Fig — Shown is an example of the results from one experimental replicate. A logarithmic plot of the gated cells is shown using FITC-A (green fluorescence, x-axis) and PE-A (area of the pulse width, red fluorescence y-axis). Autofluorescence is detected on the diagonal, where cells showing increased green fluorescence are to the right of the diagonal. Autofluorescence are gated to determine the percentage of GFP+ cells (blue). In this example the cell line U2Os with the DR-GFP cassette was used to quantify homology-directed repair (HR). The examples shown are: a. untransfected untreated control to correct for background; b. I-SceI transfected no treatment control (used as the baseline or “0%”); c-f. I-SceI transfected with different heavy metal treatments (100–500 μM NiCl2 and 1 μM CdCl2). Note that 100 μM NiCl2 increases the signal relative to the control (b), while 500 μM NiCl2 decreases the signal. (PDF) [file pone.0151367.s001.pdf]

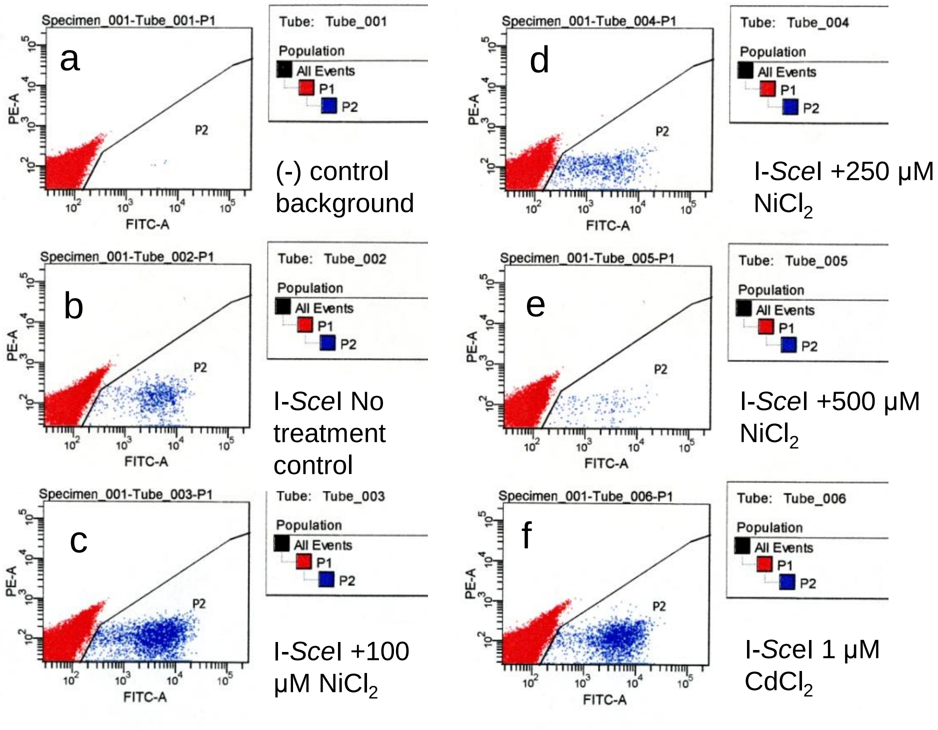

Supplemental Figure S1. Representative FACS profiles of the U2OS DR-GFP cell line. Shown is an example of the results from one experimental replicate. A logarithmic plot of the gated cells is shown using FITC-A (green fluorescence, x-axis) and PE-A (area of the pulse width, red fluorescence y-axis). Autofluorescence is detected on the diagonal, where cells showing increased green fluorescence are to the right of the diagonal. Autofluorescence are gated to determine the percentage of GFP+ cells (blue). In this example the cell line U2Os with the DR-GFP cassette was used to quantify homology-directed repair (HR). The examples shown are: **a**. untransfected untreated control to correct for background; **b**. I-*Sce*I transfected no treatment control (used as the baseline or “0 %”); **c-f**. I-*Sce*I transfected with different heavy metal treatments (100-500 μM $NiCl_2$ and 1 μM $CdCl_2$). Note that 100 μM $NiCl_2$ increases the signal relative to the control (**b**), while 500 μM $NiCl_2$ decreases the signal.
