# Supplementary material for: Heavy Metal Exposure Influences Double Strand Break DNA Repair Outcomes
Source: PLoS One. 2016 Mar 11;11(3):e0151367. doi: 10.1371/journal.pone.0151367 (PMC4788447; doi:10.1371/journal.pone.0151367)
Supplement: S2 Fig — Neutral comet assay was performed for the detection of DNA breaks of cells after a 24 h heavy metal exposure. Gamma-irradiated cells were used as a positive control. Significant differences were observed for all treatments relative to the no treatment (no Tx) control [* P < 0.05** P < 0.001, Student’s paired T-test]. (PDF) [file pone.0151367.s002.pdf]

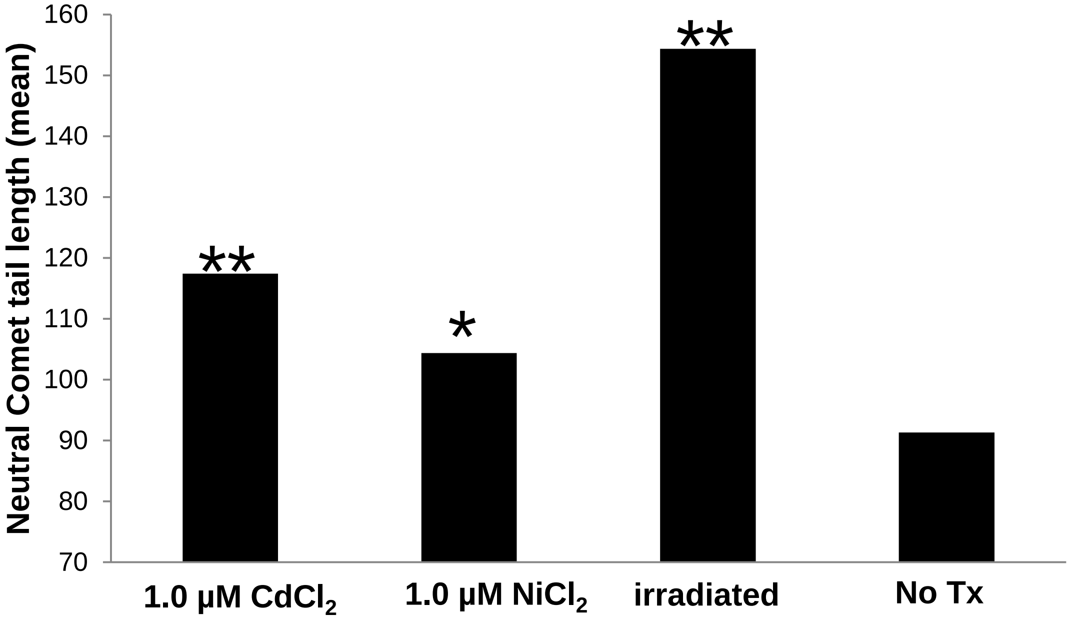

Supplemental Figure S2. Cadmium and nickel treatments induce DNA breaks. Neutral comet assay was performed for the detection of DNA breaks of cells after a 24 h heavy metal exposure. Gamma-irradiated cells were used as a positive control. Significant differences were observed for all treatments relative to the no treatment (no Tx) control [* $P < 0.05$** $P < 0.001$, Student's paired T-test].
