# Supplementary material for: Heavy Metal Exposure Influences Double Strand Break DNA Repair Outcomes
Source: PLoS One. 2016 Mar 11;11(3):e0151367. doi: 10.1371/journal.pone.0151367 (PMC4788447; doi:10.1371/journal.pone.0151367)
Supplement: S3 Fig — Shown are the examples for nickel and cadmium exposure on the four U2OS cell lines. A logarithmic plot of the gated cells is shown using FITC-A (green fluorescence, x-axis) and PE-A (area of the pulse width, red fluorescence y-axis). Autofluorescence is detected on the diagonal, where cells showing increased green fluorescence are to the right of the diagonal. Autofluorescence are gated to determine the percentage of GFP+ cells (blue). In this example the U2Os cell lines with the four different GFP cassettes were evaluated. The examples shown are: a. untransfected untreated control to correct for background; b. I-SceI transfected no treatment control; c-.treatment with 100 μM NiCl2 and d. treatment with 1 μM. Exposure to the metals alone is not sufficient to induce a GFP signal above the background in any of the four different cells lines. (PDF) [file pone.0151367.s003.pdf]

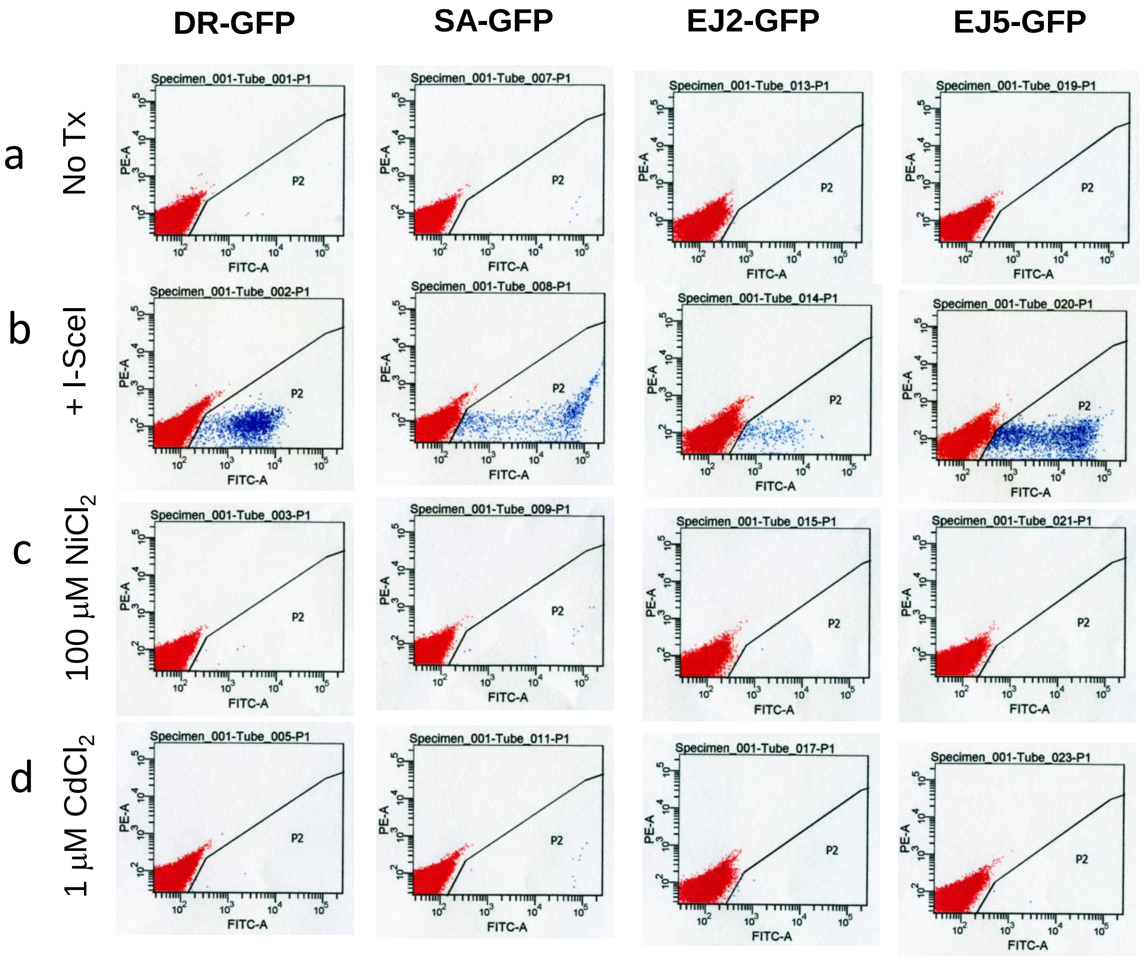

Supplemental Figure S3. The metal doses evaluated did not induce signals in the U2Os DNA repair cell line assay. Shown are the examples for nickel and cadmium exposure on the four U2OS cell lines. A logarithmic plot of the gated cells is shown using FITC-A (green fluorescence, x-axis) and PE-A (area of the pulse width, red fluorescence y-axis). Autofluorescence is detected on the diagonal, where cells showing increased green fluorescence are to the right of the diagonal. Autofluorescence are gated to determine the percentage of GFP+ cells (blue). In this example the U2Os cell lines with the four different GFP cassettes were evaluated. . The examples shown are: **a**. untransfected untreated control to correct for background; **b**. I-*Sce*I transfected no treatment control; **c-**.treatment with 100 μM $NiCl_2$ and **d**. treatment with 1 μM. Exposure to the metals alone is not sufficient to induce a GFP signal above the background in any of the four different cells lines.
