# Supplementary material for: Heavy Metal Exposure Influences Double Strand Break DNA Repair Outcomes
Source: PLoS One. 2016 Mar 11;11(3):e0151367. doi: 10.1371/journal.pone.0151367 (PMC4788447; doi:10.1371/journal.pone.0151367)
Supplement: S4 Fig — To evaluate toxicity the 0%AARP HEK cells were transfected with a plasmid expressing neomycin resistance (pIRES-EGFP, Life Technologies) and grown in the presence of 1 μM CdCl2, 1 μM AsO3,100 μM or NiCl2 for 48 h. The metal containing medium was removed and cells were grown for two weeks under G418 selection. Cell colonies were fixed and stained for 30 minutes with crystal violet (0.2% crystal violet in 5% acetic acid and 2.5% isopropanol) and counted. No significant decrease in colony numbers (P>0.05) was observed between untreated cells and heavy metal exposed cells. (PDF) [file pone.0151367.s004.pdf]

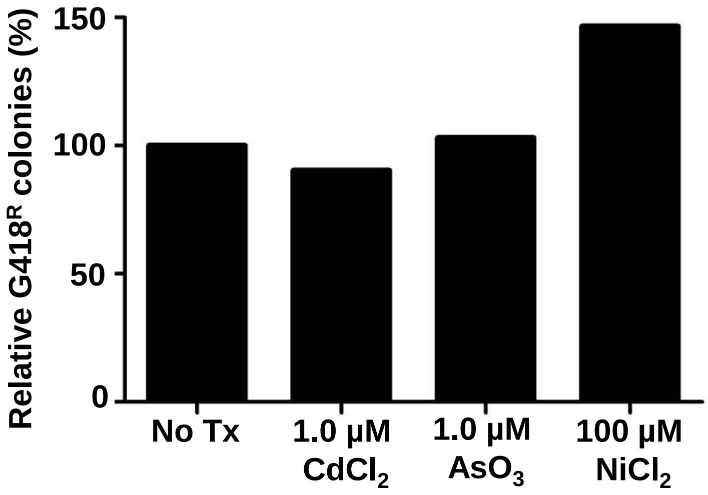

Supplemental Figure S4. The heavy metal treatments evaluated have no adverse effect on cell growth and ability to form cell colonies under selection media. To evaluate toxicity the 0%AARP HEK cells were transfected with a plasmid expressing neomycin resistance (pIRES-EGFP, Life Technologies) and grown in the presence of 1 µM $CdCl_{2,}$ 1 µM AsO_3,100 µM or $NiCl_2$ for 48 h. The metal containing medium was removed and cells were grown for two weeks under G418 selection. Cell colonies were fixed and stained for 30 minutes with crystal violet (0.2% crystal violet in 5% acetic acid and 2.5% isopropanol) and counted. No significant decrease in colony numbers (P>0.05) was observed between untreated cells and heavy metal exposed cells.
