# Supplementary material for: Heavy Metal Exposure Influences Double Strand Break DNA Repair Outcomes
Source: PLoS One. 2016 Mar 11;11(3):e0151367. doi: 10.1371/journal.pone.0151367 (PMC4788447; doi:10.1371/journal.pone.0151367)
Supplement: S5 Fig — A. A schematic of the basic diverged AARP cassette is shown. The construct consists of a promoter upstream of two different Alu sequences (e.g. 5% divergence between Alu1 and Alu2) separated by approximately 1.1 kb. Metal induced double strand breaks (DSBs) will occur in the cassette and throughout the genome. Repaired events deleting genomic DNA that brings the EF5 promoter upstream of the puromycin gene will confer puromycin resistance. B. Sequence divergence of the Alu elements yields lower signals on the Alu-Alu recombination system with a reduction in the total number of puroR colonies. The 5 and 15%AARP HEK cell line was incubated with media containing 100 μM NiCl2. Untreated cells were used as the reference control (no Tx). The results were expressed as mean ± SD of three biological replicates. (**P<0.001, Student’s paired t-test). (PDF) [file pone.0151367.s005.pdf]

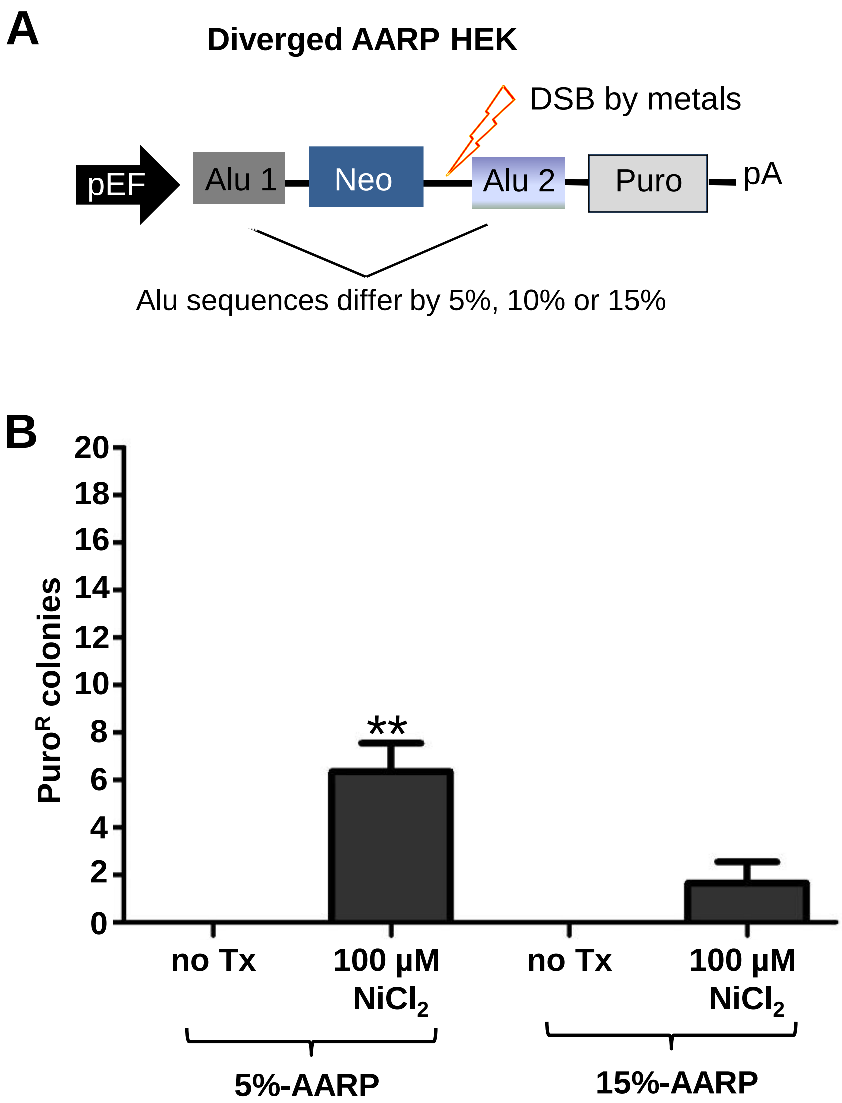

Supplemental Figure S5. **Sequence divergence of the Alu elements yields lower signals on the Alu-Alu recombination system with a reduction in the total number of puro^R colonies. A.** A schematic of the basic diverged AARP cassette is shown. The construct consists of a promoter upstream of two different Alu sequences (*e.g.* 5% divergence between Alu1 and Alu2) separated by approximately 1.1 kb. Metal induced double strand breaks (DSBs) will occur in the cassette and throughout the genome. Repaired events deleting genomic DNA that brings the EF5 promoter upstream of the puromycin gene will confer puromycin resistance. **B.** Sequence divergence of the Alu elements yields lower signals on the Alu-Alu recombination system with a reduction in the total number of puro^R colonies**.** The 5 and 15 %AARP HEK cell line was incubated with media containing 100 µM $NiCl_2$. Untreated cells were used as the reference control (no Tx). The results were expressed as mean ± SD of three biological replicates. (***P*<0.001, Student's paired t-test)
