# Supplementary material for: Heavy Metal Exposure Influences Double Strand Break DNA Repair Outcomes
Source: PLoS One. 2016 Mar 11;11(3):e0151367. doi: 10.1371/journal.pone.0151367 (PMC4788447; doi:10.1371/journal.pone.0151367)
Supplement: S6 Fig — The 5 and 10%AARP HEK cell lines were transfected with an I-SceI expression vector and incubated with media containing either A. 100 μM, NiCl2 or B. 1 μM CdCl2for 48 h before undergoing selection for 2 weeks with puromycin. Untreated cells were used as the reference control (no Tx). The results were expressed as mean ± SD of three biological replicates. (PDF) [file pone.0151367.s006.pdf]

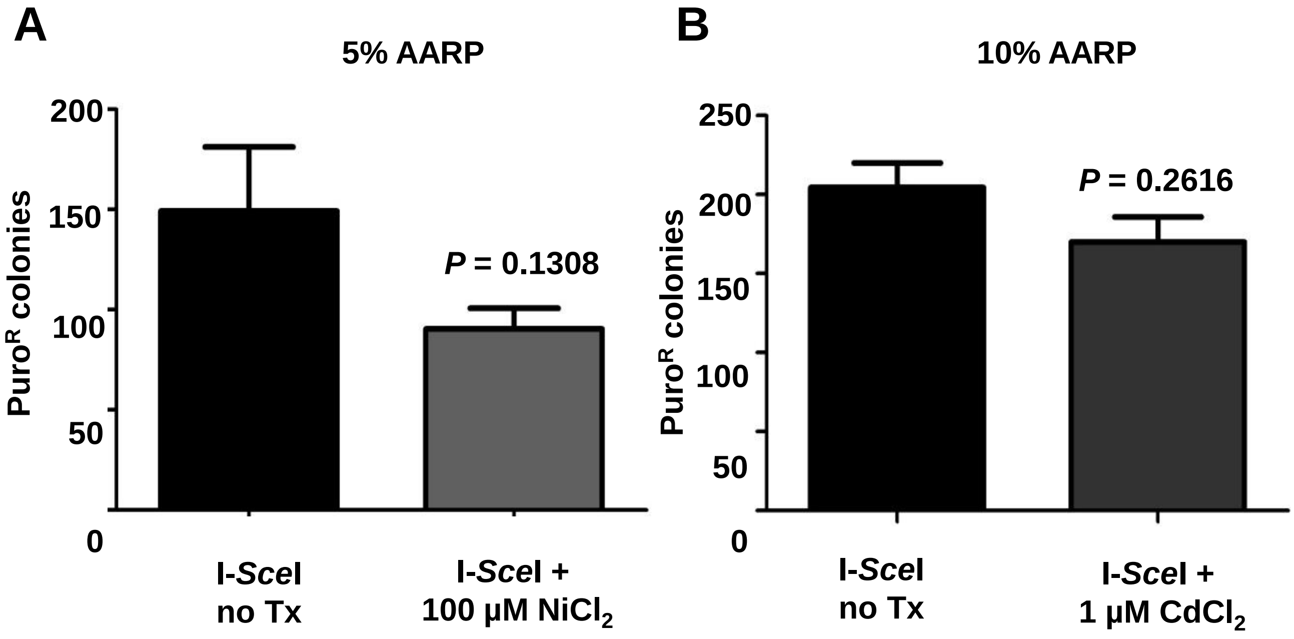

Supplemental Figure S6. Metal exposure does not increase the rate of Alu-Alu recombination signal in I-*Sce*I transfected cells. The 5 and 10%AARP HEK cell lines were transfected with an I-*Sce*I expression vector and incubated with media containing either **A.** 100 µM, $NiCl_2$ or **B.** 1 µM $CdCl_2$ for 48 h before undergoing selection for 2 weeks with puromycin. Untreated cells were used as the reference control (no Tx). The results were expressed as mean ± SD of three biological replicates.
