# Supplementary material for: Heavy Metal Exposure Influences Double Strand Break DNA Repair Outcomes
Source: PLoS One. 2016 Mar 11;11(3):e0151367. doi: 10.1371/journal.pone.0151367 (PMC4788447; doi:10.1371/journal.pone.0151367)
Supplement: S7 Fig — The specific nucleotides used to identify the Alu1-Alu2 boundaries are indicated in red for Alu2 and in blue for Alu1. A. 5% simple chimera, B. 5% complex chimera, C. 10% simple chimera and D. 10% complex chimera. (PDF) [file pone.0151367.s007.pdf]

Supplemental Figure S7. Sequence alignment of recovered of simple and complex chimera events recovered after nickel treatment. The specific nucleotides used to identify the Alu1-Alu2 boundaries are indicated in red for Alu2 and in blue for Alu1. **A.** 5% simple chimera, **B.** 5% complex chimera, **C.** 10% simple chimera and **D.** 10% complex chimera.

A. Alignment of the recovered simple chimera from 5%AARP treated with 100µM $NiCl_2$ is shown.

```
Majority     CTTAAAGGCCGGGCGCGGTGGCTCAGGCCTGTAATCCCAGCACTTAGGGAGGCCGAGGCGGGCGGTTCACGAGGTCAGGA
             ---------+---------+---------+---------+---------+---------+---------+---------+
                     170       180       190       200       210       220       230       240
             ---------+---------+---------+---------+---------+---------+---------+---------+
Alu2 5%      ------GGCCGGGCGCGGTGGCTCACGCCTGTAATCCCAGCACTTTGGGAGGCCGAGGCGGGCGGATCACGAGGTCAGGA         74
Alu1 5%      ------GGCCGGGCGCGGTGGCTCAGGCCTGTAATCCCAGCACTTAGGGAGGCCGAGGCGGGCGGTTCACGAGGTCAGGA         74
Ni3          CTTAAAGGCCGGGCGCGGTGGCTCAGGCCTGTAATCCCAGCACTTTGGGAGGCCGAGGCGGGCGGATCACGAGGTCAGGA        127
Ni4          CTTAAAGGCCGGGCGCGGTGGCTCAGGCCTGTAATCCCAGCACTTTGGGAGGCCGAGGCGGGCGGATCACGAGGTCAGGA        171
Ni12         CTTAAAGGCCGGGCGCGGTGGCTCAGGCCTGTAATCCCAGCACTTTGGGAGGCCGAGGCGGGCGGATCACGAGGTCAGGA        185
Ni13         CTTAAAGGCCGGGCGCGGTGGCTCAGGCCTGTAATCCCAGCACTTTGGGAGGCCGAGGCGGGCGGATCACGAGGTCAGGA        185
Ni10         CTTAAAGGCCGGGCGCGGTGGCTCAGGCCTGTAATCCCAGCACTTAGGGAGGCCGAGGCGGGCGGATCACGAGGTCAGGA        185
Ni11         CTTAAAGGCCGGGCGCGGTGGCTCAGGCCTGTAATCCCAGCACTTAGGGAGGCCGAGGCGGGCGGATCACGAGGTCAGGA        185
Ni9          CTTAAAGGCCGGGCGCGGTGGCTCAGGCCTGTAATCCCAGCACTTAGGGAGGCCGAGGCGGGCGGTTCACGAGGTCAGGA        185
Ni2          CTTAAAGGCCGGGCGCGGTGGCTCAGGCCTGTAATCCCAGCACTTAGGGAGGCCGAGGCGGGCGGTTCACGAGGTCAGGA        127
Ni8          CTTAAAGGCCGGGCGCGGTGGCTCAGGCCTGTAATCCCAGCACTTAGGGAGGCCGAGGCGGGCGGTTCACGAGGTCAGGA        185
Ni22         CTTAAAGGCCGGGCGCGGTGGCTCAGGCCTGTAATCCCAGCACTTAGGGAGGCCGAGGCGGGCGGTTCACGAGGTCAGGA        127
Ni5          CTTAAAGGCCGGGCGCGGTGGCTCAGGCCTGTAATCCCAGCACTTAGGGAGGCCGAGGTGGGCGGTTCACGAGGTCAGGA        140
Ni1          CTTAAAGGCCGGGCGCGGTGGCTCAGGCCTGTAATCCCAGCACTTAGGGAGGCCGAGGCGGGCGGTTCACGAGGTCAGGA        240


Majority     GATCGAGACAATCCTGGCTAACACGGTGAAACCCCGTCTCTACTAAAAATACAAAAAATTAGCCGGGCGTGGTGGCGGGC
              ---------+---------+---------+---------+---------+---------+---------+---------+
                    250       260       270       280       290       300       310       320
             ---------+---------+---------+---------+---------+---------+---------+---------+
Alu2 5%      GATCGAGACAATCCTGGCTAACACGGTGAAACCCCGTCTCTACTAAAAATACAAAAAATTAGCCGGGCGTGGTGGCGGGC        154
Alu1 5%      GATCGTGACAATCCTGGCTAACACGCTGAAACCCCGTCTCTACTATAAATACAAAAAATTAGCCGCGCGTGGTGGCGGGC        154
Ni3          GATCGAGACAATCCTGGCTAACACGGTGAAACCCCGTCTCTACTAAAAATACAAAAAATTAGCCGGGCGTGGTGGCGGGC        207
Ni4          GATCGAGACAATCCTGGCTAACACGGTGAAACCCCGTCTCTACTAAAAATACAAAAAATTAGCCGGGCGTGGTGGCGGGC        251
Ni12         GATCGAGACAATCCTGGCTAACACGGTGAAACCCCGTCTCTACTAAAAATACAAAAAATTAGCCGGGCGTGGTGGCGGGC        265
Ni13         GATCGAGACAATCCTGGCTAACACGGTGAAACCCCGTCTCTACTAAAAATACAAAAAATTAGCCGGGCGTGGTGGCGGGC        265
Ni10         GATCGAGACAATCCTGGCTAACACGGTGAAACCCCGTCTCTACTAAAAATACAAAAAATTAGCCGGGCGTGGTGGTGGGC        265
Ni11         GATCGAGACAATCCTGGCTAACACGGTGAAACCCCGTCTCTACTAAAAATACAAAAAATTAGCCGGGCGTGGTGGCGGGC        265
Ni9          GATCGAGACAATCCTGGCTAACACGGTGAAACCCCGTCTCTACTAAAAATACAAAAAATTAGCCGGGCGTGGTGGCGGGC        265
Ni2          GATCGTGACAATCCTGGCTAACACGCTGAAACCCCGTCTCTACTAAAAATACAAAAAATTAGCCGGGCGTGGTGGCGGGC        207
Ni8          GATCGTGACAATCCTGGCTAACACGCTGAAACCCCGTCTCTACTAAAAATACAAAAAATTAGCCGGGCGTGGTGGCGGGC        265
Ni22         GATCGTGACAATCCTGGCTAACACGCTGAAACCCCGTCTCTACTAAAAATACAAAAAATTAGCCGGGCGTGGTGGCGGGC        207
Ni5          GATCGTGACAATCCTGGCTAACACGCTGAAACCCCGTCTCTGCTATAAATACAAAAAATTAGCCGCGCGTGGTGGCGGGC        220
Ni1          GATCGTGACAATCCTGGCTAACACGCTGAAACCCCGTCTCTACTATAAATACAAAAAATTAGCCGCGCGTGGTGGCGGGC        320
```

```
Majority    GCCTGTAGTCCCAGCTACTCGGGAGGCTGAGGCAGGAGAATTGCGTGAACCCGGGAGGCGGAGCTTGCAGTGAGCCGAGA
            ---------+---------+---------+---------+---------+---------+---------+---------+
                   330       340       350       360       370       380       390       400
            ---------+---------+---------+---------+---------+---------+---------+---------+
Alu2 5%     GCCTGTAGTCCCAGCTACTCGGGAGGCTGAGGCAGGAGAATTGCGTGAACCCGGGAGGCGGAGCTTGCAGTGAGCCGAGA    234
Alu1 5%     GCCTGAAGTCCCAGCTACTCGGGAGCCTGAGGCAGGAGAATTGCGAGAACCCGGGAGGCGGAGCTAGCAGTGAGCCGAGA    234
Ni3         GCCTGTAGTCCCAGCTACTCGGGAGGCTGAGGCAGGAGAATTGCGTGAACCCGGGAGGCGGAGCTTGCAGTGAGCCGAGA    287
Ni4         GCCTGTAGTCCCAGCTACTCGGGAGGCTGAGGCAGGAGAATTGCGTGAACCCGGGAGGCGGAGCTTGCAGTGAGCCGAGA    331
Ni12        GCCTGTAGTCCCAGCTACTCGGGAGGCTGAGGCAGGAGAATTGCGTGAACCCGGGAGGCGGAGCTTGCAGTGAGCCGAGA    345
Ni13        GCCTGTAGTCCCAGCTACTCGGGAGGCTGAGGCAGGAGAATTGCGTGAACCCGGGAGGCGGAGCTTGCAGTGAGCCGAGA    345
Ni10        GCCTGTAGTCCCAGCTACTCGGGAGGCTGAGGCAGGAGAATTGCGTGAACCCGGGAGGCGGAGCTTGCAGTGAGCCGAGA    345
Ni11        GCCTGTAGTCCCAGCTACTCGGGAGGCTGAGGCAGGAGAATTGCGTGAACCCGGGAGGCGGAGCTTGCAGTGAGCCGAGA    345
Ni9         GCCTGTAGTCCCAGCTACTCGGGAGGCTGAGGCAGGAGAATTGCGTAAACCCGGGAGGCGGAGCTTGCAGTGAGCCGAGA    345
Ni2         GCCTGTAGTCCCAGCTACTCGGGAGGCTGAGGCAGGAGAATTGCGTGAACCCGGGAGGCGGAGCTTGCAGTGAGCCGAGA    287
Ni8         GCCTGTAGTCCCAGCTACTCGGGAGGCTGAGGCAGGAGAATTGCGTGAACCCGGGAGGCGGAGCTTGCAGTGAGCCGAGA    345
Ni22        GCCTGTAGTCCCAGCTACTCGGGAGGCTGAGGCAGGAGAATTGCGTGAACCCGGGAGGCGGAGCTTGCAGTGAGCCGAGA    287
Ni5         GCCTGTAGTCCCAGCTACTCGGGAGGCTGAGGCAGGAGAATTGCGTGAACCCGGGAGGCGGAGCTTGCAGTGAGCCGAGA    300
Ni1         GCCTGAAGTCCCAGCTACTCGGGAGCCTGAGGCAGGAGAATTGCGAGAACCCGGGAGGCGGAGCTAGCAGTGAGCCGAGA    400


Majority    TCGCGCCACTGCACTCCAGCCTGGGCGACAGAGCGAGACTCCGTCTCAAAAAAAAAAAAAAAA-GAATTCTTTTCTGCAG
            ---------+---------+---------+---------+---------+---------+---------+---------+
                   410       420       430       440       450       460       470       480
            ---------+---------+---------+---------+---------+---------+---------+---------+
Alu2 5%     TCGCGCCACTGCACTCCAGCCTGGGCGACAGAGCGAGACTCCGTCTC                                     281
Alu1 5%     TCGCGGCACTGCACTCCAGCCTGGGGGACAGAGCGAGACTCCGTCAC                                     281
Ni3         TCGCGCCACTGCACTCCAGCCTGGGCGACAGAGCGAGACTCCGTCTCAAAAAAAAAAAAAAAAA-AATTTTTTTTTGNNG    366
Ni4         TCGCGCCACTGCACTCCAGCCTGGGCGACAGAGCGAGACTCCGTCTCAAAAAAAAAAAACAAAAGAATTCTTTTCTGCAG    411
Ni12        TCGCGCCACTGCACTCCAGCCTGGGCGACAGAGCGAGACTCCGTCTCAAAAAAAAAAAAAA---GAATTCTTTTCTGCAG    422
Ni13        TCGCGCCACTGCACTCCAGCCTGGGCGACAGAGCGAGACTCCGCCTCAAAAAAAAAAAAAAAA-GAATTCTTTTCTGCAG    424
Ni10        TCGCGCCACTGCACTCCAGCCTGGGCGACAGAGCGAGACTCCGTCTCAAAAAAAAAAAAAAA--GAATTCTTTTCTGCAG    423
Ni11        TCGCGCCACTGCACTCCAGCCTGGGCGACAGAGCGAGACTCCGTCTCAAAAAAAAAAAAAAA--GAATTCTTTTCTGCAG    423
Ni9         TCGCGCCACTGCACTCCAGCCTGGGCGACAGAGCGAGACTCCGTCTCAAAAAAAAAAAAAAAAAGAATTCTTTTCTGCAG    425
Ni2         TCGCGCCACTGCACTCCAGCCTGGGCGACAGAGCGAGACTCCGTCTCAAAAAAAAAAAAAAAAA-AATTTTTTTTTNNNG    366
Ni8         TCGCGCCACTGCACTCCAGCCTGGGCGACAGAGCGAGACTCCGTCTCAAAAAAAAAAAAAAAA-GAATTCTTTTCTGCAG    424
Ni7         TCGCGGCACTGCACTCCAGCCTGGGGGACAGAGCGAGACTCCGTCACAAAAAAAAAAAAAAAA-GAATTCTTTTCTCCAG    423
Ni22        TCGCGCCACTGCACTCCAGCCTGGGCGACAGAGCGAGACTCCGTCTCAAAAAAAAAAAAAAAAAAATTTTTTTTTNNNGG    367
Ni5         TCGCGCCACTGCACTCCAGCCTGGGCGACAGAGCGAGACTCCGTCTCAAAAAAAAAAAA--------TTCTTTTCTGCAG    372
Ni1         TCGCGGCACTGCACTCCAGCCTGGGGGACAGAGCGAGACTCCGTCTCAAAAAAAAAAAAAAAAA-AATTTTTTTTTGNNG    479
```

B. Alignment of the recovered complex chimera from 5%AARP treated with 100µM $NiCl_2$ is shown.

```
Majority     GCTTAAAGGCCGGGCGCGGTGGCTCAGGCCTGTAATCCCAGCACTTXGGGAGGCCGAGGCGGGCGGTTCACGAGGTCAGG
             ---------+---------+---------+---------+---------+---------+---------+---------+
                    170       180       190       200       210       220       230       240
             ---------+---------+---------+---------+---------+---------+---------+---------+
Alu2 5%      -------GGCCGGGCGCGGTGGCTCACGCCTGTAATCCCAGCACTTTGGGAGGCCGAGGCGGGCGGATCACGAGGTCAGG         73
Alu1 5%      -------GGCCGGGCGCGGTGGCTCAGGCCTGTAATCCCAGCACTTAGGGAGGCCGAGGCGGGCGGTTCACGAGGTCAGG         73
Ni14         GCTTAAAGGCCGGGCGCGGTGGCTCAGGCCTGTAATCCCAGCACTTAGGGAGGCCGAGGCGGGCGGTTCACGAGGTCAGG        240
Ni7          GCTTAAAGGCCGGGCGCGGTGGCTCAGGCCTGTAATCCCAGCACTTAGGGAGGCCGAGGCGGGCGGTTCACGAGGTCAGG        184
Ni6          GCTTAAAGGCCGGGCGCGGTGGCTCAGGCCTGTAATCCCAGCACTTAGGGAGGCCGAGGCGGGCGGTTCACGAGGTCAGG        184
Ni15         GCTTAAAGGCCGGGCGCGGTGGCTCAGGCCTGTAATCCCAGCACTTAGGGAGGCCGAGGCGGGCGGTTCACGAGGTCAGG        240
Ni16         GCTTAAAGGCCGGGCGCGGTGGCTCAGGCCTGTAATCCCAGCACTTAGGGAGGCCGAGGCGGGCGGTTCACGAGGTCAGG        126
Ni17         GCTTAAAGGCCGGGCGCGGTGGCTCAGGCCTGTAATCCCAGCACTTTGGGAGGCCGAGGCGGGCGGTTCACGAGGTCAGG        126
Ni18         GCTTAAAGGCCGGGCGCGGTGGCTCAGGCCTGTAATCCCAGCACTTTGGGAGGCCGAGGCGGGCGGATCACGAGGTCAGG        170
Ni20         GCTTAAAGGCCGGGCGCGGTGGCTCAGGCCTGTAATCCCAGCACTTTGGGAGGCCGAGGCGGGCGGTTCACGAGGTCAGG        170
Ni21         GCTTAAAGGCCGGGCGCGGTGGCTCAGGCCTGTAATCCCAGCACTTAGGGAGGCCGAGGCGGGCGGATCACGAGGTCAGG        126
Ni23         GCTTAAAGGCCGGGCGCGGTGGCTCAGGCCTGTAATCCCAGCACTTTGGGAGGCCGAGGCGGGCGGTTCACGAGGTCAGG        130
Ni24         GCTTAAAGGCCGGGCGCGGTGGCTCAGGCCTGTAATCCCAGCACTTAGGGAGGCCGAGGCGGGCGGATCACGAGGTCAGG        130
Ni19         GCTTAAAGGCCGGGCGCGGTGGCTCAGGCCTGTAATCCCAGCACTTTGGGAGGCCGAGGCGGGCGGATCACGAGGTCAGG        170
Ni25         GCTTAAAGGCCGGGCGCGGTGGCTCAGGCCTGTAATCCCAGCACTTTGGGAGGCCGAGGCGGGCGGATCACGAGGTCAGG        130

Majority     AGATCGAGACAATCCTGGCTAACACGXTGAAACCCCGTCTCTACTAAAAATACAAAAAATTAGCCGXGCGTGGTGGCGGG
             ---------+---------+---------+---------+---------+---------+---------+---------+
                    250       260       270       280       290       300       310       320
             ---------+---------+---------+---------+---------+---------+---------+---------+
Alu2 5%      AGATCGAGACAATCCTGGCTAACACGGTGAAACCCCGTCTCTACTAAAAATACAAAAAATTAGCCGGGCGTGGTGGCGGG        153
Alu1 5%      AGATCGTGACAATCCTGGCTAACACGCTGAAACCCCGTCTCTACTATAAATACAAAAAATTAGCCGCGCGTGGTGGCGGG        153
Ni14         AGATCGTGACAATCCTGGCTAACACGCTGAAACCCCGTCTCTACTAAAAATACAAAAAATTAGCCGCGCGTGGTGGCGGG        320
Ni7          AGATCGTGACAATCCTGGCTAACACGCTGAAACCCCGTCTCTACTATAAATACAAAAAATTAGCCGGGCGTGGTGGCGGG        263
Ni6          AGATCGTGACAATCCTGGCTAACACGCTGAAACCCCGTCTCTACTAAAAATACAAAAAATTAGCCGGGCGTGGTGGCGGG        263
Ni15         AGATCGAGACAATCCTGGCTAACACGGTGAAACCCCGTCTCTACTAAAAATACAAAAAATTAGCCGGGCGTGGTGGCGGG        320
Ni16         AGATCGAGACAATCCTGGCTAACACGGTGAAACCCCGTCTCTACTATAAATACAAAAAATTAGCCGCGCGTGGTGGCGGG        206
Ni17         AGATCGTGACAATCCTGGCTAACACGGTGAAACCCCGTCTCTACTAAAAATACAAAAAATTAGCCGGGCGTGGTGGCGGG        206
Ni18         AGATCGAGACAATCCTGGCTAACACGCTGAAACCCCGTCTCTACTATAAATACAAAAAATTAGCCGGGCGTGGTGGCGGG        250
Ni20         AGATCGTGACAATCCTGGCTAACACGCTGAAACCCCGTCTCTACTATAAATACAAAAAATTAGCCGCGCGTGGTGGCGGG        250
Ni21         AGATCGTGACAATCCTGGCTAACACGGTGAAACCCCGTCTCTACTAAAAATACAAAAAATTAGCCGCGCGTGGTGGCGGG        206
Ni23         AGATCGAGACAATCCTGGCTAACACGCTGAAACCCCGTCTCTACTAAAAATACAAAAAATTAGCCGCGCGTGGTGGCGGG        210
Ni24         AGATCGAGACAATCCTGGCTAACACGCTGAAACCCCGTCTTTACTAAAAATACAAAAAATTAGCCGCGCGTGGTGGCGGG        210
Ni19         AGATCGAGACAATCCTGGCTAACACGGTGAAACCCCGTCTCTACTAAAAATACAAAAAATTAGCCGGGCGTGGTGGCGGG        250
Ni25         AGATCGAGACAATCCTGGCTAACACGGTGAAACCCCGTCTCTACTAAAAATACAAAAAATTAGCCGCGCGTGGTGGCGGG        210
```

```
Majority     CGCCTGTAGTCCCAGCTACTCGGGAGGCTGAGGCAGGAGAATTGCGTGAACCCGGGAGGCGGAGCTTGCAGTGAGCCGAG
             ---------+---------+---------+---------+---------+---------+---------+---------+
                    330       340       350       360       370       380       390       400
             ---------+---------+---------+---------+---------+---------+---------+---------+
Alu2 5%      CGCCTGTAGTCCCAGCTACTCGGGAGGCTGAGGCAGGAGAATTGCGTGAACCCGGGAGGCGGAGCTTGCAGTGAGCCGAG      233
Alu1 5%      CGCCTGAAGTCCCAGCTACTCGGGAGCCTGAGGCAGGAGAATTGCGAGAACCCGGGAGGCGGAGCTAGCAGTGAGCCGAG      233
Ni14         CGCCTGAAGTCCCAGCTACTCGGGAGCCTGAGGCAGGAGAATTGCGAGAACCCGGGAGGCGGAGCTAGCAGTGAGCCGAG      400
Ni7          CGCCTGAAGTCCCAGCTACTCGGGAGCCTGAGGCAGGAGAATTGCGAGAACCCGGGAGGCGGAGCTAGCAGTGAGCCGAG      343
Ni6          CGCCTGAAGTCCCAGCTACTCGGGAGCCTGAGGCAGGAGAATTGCGAGAACCCGGGAGGCGGAGCTAGCAGTGAGCCGAG      343
Ni15         CGCCTGTAGTCCCAGCTACTCGGGAGGCTGAGGCAGGAGAATTGCGAGAACCCGGGAGGCGGAGCTAGCAGTGAGCCGAG      400
Ni16         CGCCTGTAGTCCCAGCTACTCGGGAGGCTGAGGCAGGAGAATTGCGTGAACCCGGGAGGCGGAGCTTGCAGTGAGCCGAG      286
Ni17         CGCCTGTAGTCCCAGCTACTCGGGAGGCTGAGGCAGGAGAATTGCGTGAACCCGGGAGGCGGAGCTTGCAGTGAGCCGAG      286
Ni18         CGCCTGTAGTCCCAGCTACTCGGGAGGCTGAGGCAGGAGAATTGCGTGAACCCGGGAGGCGGAGCTTGCAGTGAGCCGAG      330
Ni20         CGCCTGTAGTCCCAGCTACTCGGGAGCCTGAGGCAGGAGAATTGCGAGAACCCGGGAGGCGGAGCTAGCAGTGAGCCGAG      330
Ni21         CGCCTGTAGTCCCAGCTACTCGGGAGGCTGAGGCAGGAGAATTGCGTGAACCCGGGAGGCGGAGCTTGCAGTGAGCCGAG      286
Ni23         CGCCTGTAGTCCCAGCTACTCGGGAGCCTGAGGCAGGAGAATTGCGTGAACCCGGGAGGCGGAGCTAGCAGTGAGCCGAG      290
Ni24         CGCCTGAAGTCCCAGCTACTCGGGAGGCTGAGGCAGGAGAATTGCGTGAACCCGGGAGGCGGAGCTTGCAGTGAGCCGAG      290
Ni19         CGCCTGTAGTCCCAGCTACTCGGGAGGCTGAGGCAGGAGAATTGCGTGAACCCGGGAGGCGGAGCTTGCAGTGAGCCGAG      330
Ni25         CGCCTGAAGTCCCAGCTACTCGGGAGCCTGAGGCAGGAGAATTGCGTGAACCCGGGAGGCGGAGCTTGCAGTGAGCCGAG      290

Majority     ATCGCGCCACTGCACTCCAGCCTGGGCGACAGAGCGAGACTCCGTCTCAAAAAAAAAAAAAAAAAAATTTTTTTTTGXXG
             ---------+---------+---------+---------+---------+---------+---------+---------+
                    410       420       430       440       450       460       470       480
             ---------+---------+---------+---------+---------+---------+---------+---------+
Alu2 5%      ATCGCGCCACTGCACTCCAGCCTGGGCGACAGAGCGAGACTCCGTCTC                                     281
Alu1 5%      ATCGCGGCACTGCACTCCAGCCTGGGGGACAGAGCGAGACTCCGTCAC                                     281
Ni14         ATCGCGGCACTGCACTCCAGCCTGGGGGACAGAGCGAGACTCCGTCACAAAAAAAAAAAAAAAAAANTTTTTTTTGGNGG      480
Ni7          ATCGCGGCACTGCACTCCAGCCTGGGGGACAGAGCGAGACTCCGTCACAAAAAAAAAAAAAAAA-GAATTCTTTTCTCCA      422
Ni6          ATCGCGGCACTGCACTCCAGCCTGGGCGACAGAGCGAGACTCCGTCTCAAAAAAAAAAAAAAAAAGAATTCTTTTCTGCA      422
Ni15         ATCGCGCCACTGCACTCCAGCCTGGGCGACAGAGCGAGACTCCGTCTCAAAAAAAAAAAAAAAAAAATTTTTTTTTGNNG      480
Ni16         ATCGCGCCACTGCACTCCAGCCTGGGCGACAGAGCGAGACTCCGTCTCAAAAAAAAAAAAAAAAAATTTTTTTTTTGNNG      366
Ni17         ATCGCGCCACTGCACTCCAGCCTGGGCGACAGAGCGAGACTCCGTCTCAAAAAAAAAAAAAAAAAATTTTTTTTTTGNNG      366
Ni18         ATCGCGCCACTGCACTCCAGCCTGGGCGACAGAGCGAGACTCCGTCTCAAAAAAAAAAAAAAAAGAATTCTTTTCTGCAG      410
Ni19         ATCGCGCCACTGCACTCCAGCCTGGGGGACAGAGCGAGACTCCGTTTCAAAAAAAAAAAAAAAAGAATT-TTTTNNGCAG      409
Ni20         ATCGCGGCACTGCACTCCAGCCTGGGGGACAGAGCGAGACTCCGTCTCAAAAAAAAAAAAAAAGAA-TTCTTTTCTGCAG      409
Ni21         ATCGCGCCACTGCACTCCAGCCTGGGCGACAGAGCGAGACTCCGTCTCAAAAAAAAAAAAAAAAAATTTTTTTTTTGNNG      366
Ni22         ATCGCGCCACTGCACTCCAGCCTGGGCGACAGAGCGAGACTCCGTCACAAAAAAAAAAAAAAAAAAATTTTTTTTTNNNG      366
Ni23         ATCGCGCCACTGCACTCCAGCCTGGGCGACAGAGCGAGACTCCGTCTCAAAAAAAAAAAAAAAAG----NTNNN           360
Ni24         ATCGCGCCACTGCACTCCAGCCTGGGCGACAGAGCGAGACTCCGTCTCNNNNNNNNANNNNNNAAANNN                360
Ni19         ATCGCGCCACTGCACTCCAGCCTGGGGGACAGAGCGAGACTCCGTTTCAAAAAAAAAAAAAAAAGAATT-TTTTNNGCAG      409
Ni25         ATCGCGCCACTGCACTCCAGCCTGGGCGACAGAGCGAGACTCCGTCTCAAAAAAAAAAAAAAAG----------------      354
```

C. Alignment of the recovered simple chimera from 10%AARP treated with 100µM $NiCl_2$ is shown.

```
 Majority     CAGGCTTGGTACCGAGAAGCTTAAAGGCCGGGCGTGGTGGCTCAGGCCTGTAATGCCAGCACTTAGGGAGGCCGAGGCGG
             ---------+---------+---------+---------+---------+---------+---------+---------+
                      90        100       110       120       130       140       150       160
             ---------+---------+---------+---------+---------+---------+---------+---------+
Alu2 10%     -------------------------GGCCGGGCGCGGTGGCTCACGCCTGTAATCCCAGCACTTTGGGAGGCCGAGGCGG          55
Alu1 10%     -------------------------GGCCGGGCGTGGTGGCTCAGGCCTGTAATGCCAGCACTTAGGGAGGCCGTGGCGG          55
Ni34         CAGGCTTGGTACCGAGAAGCTTAAAGGCCGGGCGTGGTGGCTCAGGCCTGTAATGCCAGCACTTAGGGAGGCCGTGGCGG          90
Ni26         CAGGCTTGGTACCGAGAAGCTTAAAGGCCGGGCGTGGTGGCTCAGGCCTGTAATGCCAGCACTTAGGGAGGCCGCGGCGG         160
Ni29         CAGGCTTGGTACCGAGAAGCTTAAAGGCCGGGCGTGGTGGCTCAGGCCTGTAATGCCAGCACTTAGGGAGGCCGTGGCGG          90
Ni32         CAGGCTTGGTACCGAGAAGCTTAAAGGCCGGGCGTGGTGGCTCAGGCCTGTAATCCCAGCACTTTGGGAGGCCGAGGCGG          90
Ni30         CAGGCTTGGTACCGAGAAGCTTAAAGGCCGGGCGTGGTGGCTCAGGCCTGTAATCCCAGCACTTTGGGAGGCCGAGGCGG          90
Ni42         CAGGCTTGGTACCGAGAAGCTTAAAGGCCGGGCGTGGTGGCTCAGGCCTGTAATCCCAGCACTTTGGGAGGCCGAGGCGG          99
Ni27         CAGGCTTGGTACCGAGAAGCTTAAAGGCCGGGCGCGGTGGCTCACGCCTGTAATCCCAGCACTTTGGGAGGCCGAGGCGG          90
Ni28         CAGGCTTGGTACCGAGAAGCTTAAAGGCCGGGCGCGGTGGCTCACGCCTGTAATCCCAGCACTTTGGGAGGCCGAGGCGG         160

Majority     GCGGATCACGAGGTCAGGAGATCGAGACAATCCTGGCTAACACGGTGAAACCCCGTCTCTACTAAAAATACAAAAAATTA
             ---------+---------+---------+---------+---------+---------+---------+---------+
                      170       180       190       200       210       220       230       240
             ---------+---------+---------+---------+---------+---------+---------+---------+
Alu2 10%     GCGGATCACGAGGTCAGGAGATCGAGACAATCCTGGCTAACACGGTGAAACCCCGTCTCTACTAAAAATACAAAAAATTA         135
Alu1 10%     GCGGTTCACGAGGTGAGGAGATCGTGACAATCCTCGCTAACACGCTGAAACCCTGTCTCTACTATAAATACAAATAATTA         135
Ni34         GCGGTTCACGAGGTGAGGAGATCGTGACAATCCTCGCTAACACGCTGAAACCCTGTCTCTACTATAAATACAAATAATTA         170
Ni26         GCGGTTCACGAGGTGAGGAGATCGTGACAATCCTCGCTAACACGCTGAAACCCTGTCTCTACTATAAATACAAATAATTA         240
Ni29         GCGGATCACGAGGTCAGGAGATCGAGACAATCCTGGCTAACACGGTGAAACCCCGTCTCTACTAAAAATACAAAAAATTA         170
Ni32         GCGGATCACGAGGTCAGGAGATCGAGACAATCCTGGCTAACACGGTGAAACCCCGTCTCTACTAAAAATACAAAAAATTA         170
Ni30         GCGGATCACGAGGTCAGGAGATCGAGACAATCCTGGCTAACACGGTGAAACCCCGTCTCTAGNAAAAATACAAAAAATTA         170
Ni42         GCGGATCACGAGGTCAGGAGATCGAGACAATCCTGGCTAACACGGTGAAACCCCGTCTCTACTAAAAATACAAAAAATTA         179
Ni27         GCGGATCACGAGGTCAGGAGATCGAGACAATCCTGGCTAACACGGTGAAACCCCGTCTCTACTAAAAATACAAAAAATTA         170
Ni28         GCGGATCACGAGGTCAGGAGATCGAGACAATCCTGGCTAACACGGTGAAACCCCGTCTCTACTAAAAATACAAAAAATTA         240


Majority     GCCGGGCGTGGTGGCGGGCGCCTGTAGTCCCAGCTACTCGGGAGGCTGAGGCAGGAGAATTGCGTGAACCCGGGAGGCGG
             ---------+---------+---------+---------+---------+---------+---------+---------+
                      250       260       270       280       290       300       310       320
             ---------+---------+---------+---------+---------+---------+---------+---------+
Alu2 10%     GCCGGGCGTGGTGGCGGGCGCCTGTAGTCCCAGCTACTCGGGAGGCTGAGGCAGGAGAATTGCGTGAACCCGGGAGGCGG         215
Alu1 10%     GCCGCGCGTGGTGGTGGGCGCCTGAAGTCCCAGCAACTCGGGAGCCTGAGGCAGCAGAATTGCGAGAACCCGGGTGGCGG         215
Ni34         GCCGCGCGTGGTGGTGGGCGCCTGAAGTCCCAGCAACTCGGGAGCCTGAGGCAGCAGAATTGCTGGAACCCGGGTGGCGG         250
Ni26         GCCGCGCGTGGTGGTGGGCGCCTGAAGTCCCAGCAACTCGGGAGCCTGAGGCAGCAGAATTGCGAGAACCCGGGTGGCGG         320
Ni29         GCCGGGCGTGGTGGCGGGCGCCTGTAGTCCCAGCTACTCGGGAGGCTGAGGCAGGAGAATTGCGTGAACCCGGGAGGCGG         250
Ni32         GCCGGGCGTGGTGGCGGGCGCCTGTAGTCCCAGCTACTCGGGAGGCTGAGGCAGGAGAATTGCGTGAACCCGGGAGGCGG         250
Ni30         NCCGGGCGTGGTGGCGGGCGCCTGTAGTCCCAGCTACTCGGGAGGCTGAGGCAGGAGAATTGCGTGAACCCGGGAGGCGG         250
Ni42         GCCGGGCGTGGTGGCGGGCGCCTGTAGTCCCAGCTACTCGGGAGGCTGAGGCAGGAGAATTGCGTGAACCCGGGAGGCGG         259
Ni27         GCCGGGCGTGGTGGCGGGCGCCTGTAGTCCCAGCTACTCGGGAGGCTGAGGCAGGAGAATTGCGTGAACCCGGGAGGCGG         250
Ni28         GCCGGGCGTGGTGGCGGGCGCCTGTAGTCCCAGCTACTCGGGAGGCTGAGGCAGGAGAATTGCGTGAACCCGGGAGGCGG         320
```

```
Majority     AGCTTGCAGTGAGCCGAGATCGCGCCACTGCACTCCAGCCTGGGCGACAGAGCGAGACTCCGTCTCAAAAAAAAAAAAAA
             ---------+---------+---------+---------+---------+---------+---------+---------+
                    330       340       350       360       370       380       390       400
             ---------+---------+---------+---------+---------+---------+---------+---------+
Alu2 10%     AGCTTGCAGTGAGCCGAGATCGCGCCACTGCACTCCAGCCTGGGCGACAGAGCGAGACTCCGTCTCAAAAAAAAAAAAAA        295
Alu1 10%     AGCTAGCAGTGAGCGGAGATCGCGGCACTGCACTGCAGCCTGGGGGACAGAGCGTGACTCCGTCACAAAAAAAATAAAAA        295
Ni34         AGCTAGCANNGAGCGGAGATCGCGGCACTGCNNTGCAGCCTGGGGGACANAACGTGACTCCGTCACAAAAAAAATAAAA        330
Ni26         AGCTAGCAGTGAGCGGAGATCGCGGCACTGCACTGCAGCCTGGGCGACAGAGCGAGACTCCGTCTCAAAAAAAAAAAAAA        400
Ni29         AGCTTGCAGTGAGCCGAGATCGCGCCACTGCACTCCAGCCTGGGCGACAGAGNGAGACTCCGTCTCAAATAAAAAAAAAA        330
Ni32         AGCTTGCATTGAGCCGAGATCGCGCCACTGCACTCCAGCCTGGGCGACAGAGCGAGACTCCGTCTCAAAAAAAAAAAAAA        330
Ni30         AGCTTGCAGTGANCCGAGATCGCGCCACTGCACTCCAGCCTGGGCGACAGAGCGAGACTCCGTCTCAAAAAAAAAAAAAA        330
Ni42         AGCTTGCAGTGAGCCGAGATCACGCCACTGCACTCCAGCCTGGGCGACAGAGCGAGACTCCGTCTCAAAAAAAAAAAAAA        339
Ni27         AGCTTGCAGTGAGCCGAGATCGCGCCACTGCACTCCAGCCTGGGCGACAGAGCGAGACTCCGTCTCAAAAAAAAAAAAAA        330
Ni28         AGCTTGCAGTGAGCCGAGATCGCGCCACTGCACTCCAGCCTGGGCGACAGAGCGAGACTCCGTCTCAAAAAAAAAAAAAA        400
```

D. Alignment of the recovered complex chimera from 10%AARP treated with 100µM $NiCl_2$ is shown.

```
Majority     CAGGCTTGGTACCGAGAAGCTTAAAGGCCGGGCGTGGTGGCTCAGGCCTGTAATGCCAGCACTTAGGGAGGCCGXGGCGG
             ---------+---------+---------+---------+---------+---------+---------+---------+
                     90       100       110       120       130       140       150       160
             ---------+---------+---------+---------+---------+---------+---------+---------+
Alu2 10%     -------------------------GGCCGGGCGCGGTGGCTCACGCCTGTAATCCCAGCACTTTGGGAGGCCGAGGCGG         55
Alu1 10%     -------------------------GGCCGGGCGTGGTGGCTCAGGCCTGTAATGCCAGCACTTAGGGAGGCCGTGGCGG         55
Ni31         CAGGCTTGGTACCGAGAAGCTTAAAGGCCGGGCGTGGTGGCTCAGGCCTGTAATGCCAGCACTTTGGGAGGCCGAGGCGG         90
Ni35         CAGGCTTGGTACCGAGAAGCTTAAAGGCCGGGCGTGGTGGCTCAGGCCTGTAATGCCAGCACTTAGGGAGGCCGTGGCGG        160
Ni36         CAGGCTTGGTACCGAGAAGCTTAAAGGCCGGGCGTGGTGGCTCAGGCCTGTAATGCCAGCACTTAGGGAGGCCGTGGCGG         90
Ni37         CAGGCTTGGTACCGAGAAGCTTAAAGGCCGGGCGTGGTGGCTCAGGCCTGTAATCCCAGCACTTTGGGAGGCCGAGGCGG         90
Ni38         CAGGCTTGGTACCGAGAAGCTTAAAGGCCGGGCGTGGTGGCTCACGCCTGTAATCCCAGCACTTAGGGAGGCCGAGGCGG         90
Ni33         CAGGCTTGGTACCGAGAAGCTTAAAGGCCGGGCGTGGTGGCTCAGGCCTGTAATGCCAGCACTTAGGGAGGCCGAGGCGG         90
Ni39         CAGGCTTGGTACCGAGAAGCTTAAAGGCCGGGCGTGGTGGCTCAGGCCTGTAATGCCAGCACTTAGGGAGGCCGTGGCGT        121
Ni40         CAGGCTTGGTACCGAGAAGCTTAAAGGCCGGGCGTGGTGGCTCAGGCCTGTAATGCCAGCACTTTGGGAGGCCGAGGCGG        121
Ni41         CAGGCTTGGTACCGAGAAGCTTAAAGGCCGGGCGTGGTGGCTCAGGCCTGTAATGCCAGCACTTAGGGAGGCCGTGGCGG        121
```

```
Majority     GCGGXTCACGAGGTXAGGAGATCGAGACAATCCTGGCTAACACGCTGAAACCCXGTCTCTACTATAAATACAAAAAATTA
             ---------+---------+---------+---------+---------+---------+---------+---------+
                    170       180       190       200       210       220       230       240
             ---------+---------+---------+---------+---------+---------+---------+---------+
Alu2 10%     GCGGATCACGAGGTCAGGAGATCGAGACAATCCTGGCTAACACGGTGAAACCCCGTCTCTACTAAAAATACAAAAAATTA     135
Alu1 10%     GCGGTTCACGAGGTGAGGAGATCGTGACAATCCTCGCTAACACGCTGAAACCCTGTCTCTACTATAAATACAAATAATTA     135
Ni31         GCGGATCACGAGGTCAGGAGATCGAGACAATCCTGGCTAACACGGTGAAACCCCGTCTCTACTAAAAATACAAAAAATTA     170
Ni35         GCGGATCACGAGGTCAGGAGATCGAGACAATCCTGGCTAACACGCTGAAACCCTGTCTCTACTATAAATACAAATAATTA     240
Ni36         GCGGTTCACGAGGTGAGGAGATCGTGACAATCCTGGCTAACACGCTGAAACCCCGTCTCTACTATAAATACAAAAAATTA     170
Ni37         GCGGATCACGAGGTCAGGAGATCGAGACAATCCTGGCTAACACGGTGAAACCCCGTCTCTACTATAAATACAAATAATTA     170
Ni38         GCGGATCACGAGGTGAGGAGATCGAGACAATCCTGGCTAACACGGTGAAACCCCGTCTCTACTAAAAATACAAAAAATTA     170
Ni33         GCGGATCACGAGGTCAGGAGATCGAGACAATCCTGGCTAACACGGTGAAACCCCGTCTCTACTATAAATACAAATAATTA     170
Ni39         GCGGTTCACGAGGTGAGGAGATCGTGACAATCCTCGCTAACACGCTGAAACCCTGTCTCTACTATAAATACAAAAAATTA     201
Ni40         GCGGTTCACGAGGTCAGGAGATCGAGACAATCCTCGCTAACACGCTGAAACCCTGTCTCTACTAAAAATACAAAAAATTA     201
Ni41         GCGGTTCACGAGGTGAGGAGATCGTGACAATCCTCGCTAACACGCTGAAACCCTGTCTCTACTATAGATACAAATAATTA     201

Majority     GCCGCGCGTGGTGGCGGGCGCCTGTAGTCCCAGCTACTCGGGAGGCTGAGGCAGGAGAATTGCGTGAACCCGGGAGGCGG
             ---------+---------+---------+---------+---------+---------+---------+---------+
                    250       260       270       280       290       300       310       320
             ---------+---------+---------+---------+---------+---------+---------+---------+
Alu2 10%     GCCGGGCGTGGTGGCGGGCGCCTGTAGTCCCAGCTACTCGGGAGGCTGAGGCAGGAGAATTGCGTGAACCCGGGAGGCGG     215
Alu1 10%     GCCGCGCGTGGTGGTGGGCGCCTGAAGTCCCAGCAACTCGGGAGCCTGAGGCAGCAGAATTGCGAGAACCCGGGTGGCGG     215
Ni31         GCCGGGCGTGGTGGCGGGCGCCTGTAGTCCCAGCTACTCGGGAGGCTGAGGCAGCAGAATTGCGTGAACCCGGGAGGCGG     250
Ni35         GCCGCGCGTGGTGGCGGGCGCCTGTAGTCCCAGCTACTCGGGAGGCCGAGGCAGGAGAATTGCGTGAACCCGGGAGGCGG     320
Ni36         GCCGCGCGTGGTGGCGGGCGCCTGAAGTCCCAGCTACTCGGGAGGCTGAGGCAGGAGAATTGCGTGAACCCGGGAGGCGG     250
Ni37         GCCGCGCGTGGTGGTGGGCGCCTGTAGTCCCAGCTACTCGGGAGGCTGAGGCAGGAGAATTGCGTGAACCCGGGAGGCGG     250
Ni38         GCCGGGCGTGGTGGCGGGCGCCTGTAGTCCCAGCTACTCGGGAGGCTGAGGCAGGAGAATTGCGTGAACCCGGGAGGCGG     250
Ni33         GCCGCGCGTGGTGGTGGGCGCCTGTAGTCCCAGCTACTCGGGAGGCTGAGGCAGGAGAATTGCGTGAACCCGGGAGGCGG     250
Ni39         GCCGCGCGTGGTGGTGGGCGCCTGAAGTCCCAGCTACTCGGGAGCCTGAGGCAGCAGAATTGCGAGAACCCGGGTGGCGG     281
Ni40         GCCGGGCGTGGTGGCGGGCGCCTGTAGTCCCAGCTACTCGGGAGGCTGAGGCAGCANAATTGCGAGAACCCGGGTGGCGG     281
Ni41         GCCGCGCGTGGTGGTGGGCGCCTGAAGTCCCAGCAACTCGGGAGCCTGAGGCAGGAGAATTGCGTGAACCCGGGTGGCGG     281

 Majority     AGCTTGCAGTGAGCCGAGATCGCGCCACTGCACTCCAGCCTGGGCGACAGAGCGAGACTCCGTCTCAAAAAAAAAAAAAA
             ---------+---------+---------+---------+---------+---------+---------+---------+
                    330       340       350       360       370       380       390       400
             ---------+---------+---------+---------+---------+---------+---------+---------+
Alu2 10%     AGCTTGCAGTGAGCCGAGATCGCGCCACTGCACTCCAGCCTGGGCGACAGAGCGAGACTCCGTCTCAAAAAAAAAAAAAA     295
Alu1 10%     AGCTAGCAGTGAGCGGAGATCGCGGCACTGCACTGCAGCCTGGGGGACAGAGCGTGACTCCGTCACAAAAAAAATAAAAA     295
Ni31         AGCTTGCAGTGAGCCGAGATCGCGCCACTGCACTCCAGCCTGGGCGACAGAGCGAGACTCCGTCTCAAAAAAAAAAAAAA     330
Ni35         AGCTTGCAGTGAGCCGAGATCGCGCCACTGCACTCCAGCCTGGGCGACAGAGCGAGACTCCGTCTCAAAAAAAAAAAAAA     400
Ni36         AGCTTGCAGTGAGCCGAGATCGCGCCACTGCACTCCAGCCTGGGCGACAGAGCGAGACTCCGTCTCAAAAAAAAAAAAAA     330
Ni37         AGCTTGCAGTGAGCCGAGATCGCGCCACTGCACTGCAGCCTGGGCGACACAGCGAGACTCCGTCACAAAAAAAAAAAAAA     330
Ni38         AGCTTGCAGTGAGCCGAGATCGCGCCACTGCACTCCAGCCTGGGCGACAGAGCGAGACTCCGTCTCAAAAAAAAAAAAAA     330
Ni33         AGCTTGCAGTGAGCCGAGATCGCGCCACTGCACTCCAGCCTGGGCGACACAGCGAGACTCCGTCTCAAAAAAAAAAAAAA     330
Ni39         AGCTAGCAGTGAGCGGAGATCGCGCCACTGCACTCCAGCCTGGGCGACAGAGCGAGACTCCGTCTCAAAAAAAAAAAAAA     361
Ni40         AGCTAGCAGTGAGCGGAGATCGCGGCACTGCACTCCAGCCTGGGCGACAGAGCGAGACTCCGTCTCAAAAAAAAAAAAAA     361
Ni41         AGCTAGCAGTGAGCCGAGATCGCGGCACTGCACTGCAGCCTGGGCGACAGAGCGAGACTCCGTCTCAAAAAAAAAAAAAA     361
```
