# Supplementary material for: Heavy Metal Exposure Influences Double Strand Break DNA Repair Outcomes
Source: PLoS One. 2016 Mar 11;11(3):e0151367. doi: 10.1371/journal.pone.0151367 (PMC4788447; doi:10.1371/journal.pone.0151367)
Supplement: S8 Fig — ISce-I transfected 0%AARP HEK cells were incubated with media containing 1 μM CdCl2, 100 μM NiCl2, or 1 μM AsO3 following the same protocol used to generate the experimental data (Figs 3–5). Untreated cells were used as the reference control (no Tx). The cells were harvested after 48 h exposure time and analyzed with propidium iodide staining to assess cell cycle distribution by FACS analysis. Shown is an example of the results in A the raw histograms (note difference in scales) and in B the graph of the percentages of cells in each phase of the cell. (PDF) [file pone.0151367.s008.pdf]

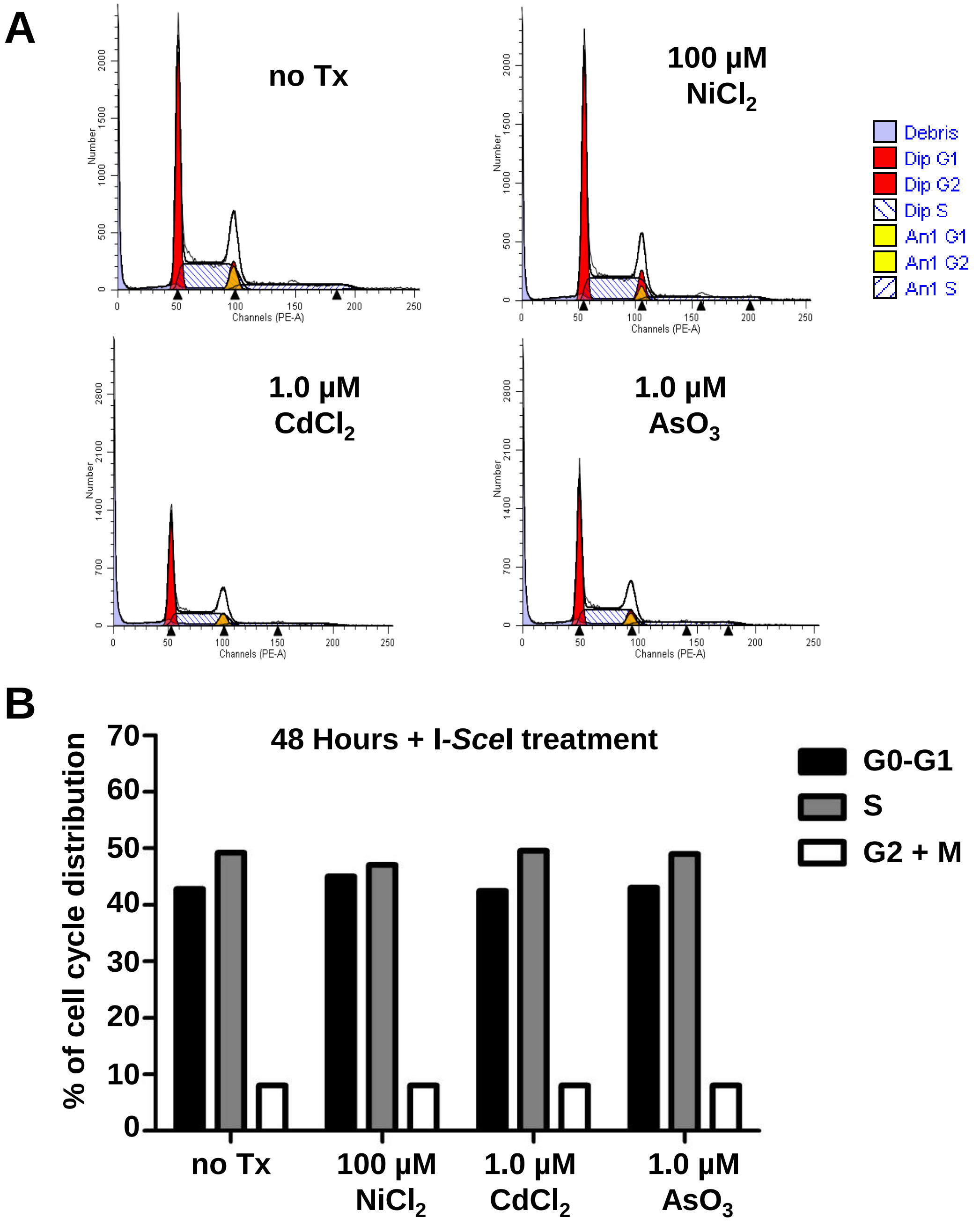

Supplemental Figure S8. The metal treatments evaluated have minimal to no effect on cell cycle. I*Sce*-I transfected 0%AARP HEK cells were incubated with media containing 1 µM $CdCl_2$, 100 µM $NiCl_2$, or 1 µM AsO_3 following the same protocol used to generate the experimental data (Figures 3-5). Untreated cells were used as the reference control (no Tx). The cells were harvested after 48 h exposure time and analyzed with propidium iodide staining to assess cell cycle distribution by FACS analysis. Shown is an example of the results in **A** the raw histograms (note difference in scales) and in **B** graph of the percentages of cells in each phase of the cell.
