# Supplementary material for: Heavy Metal Exposure Influences Double Strand Break DNA Repair Outcomes
Source: PLoS One. 2016 Mar 11;11(3):e0151367. doi: 10.1371/journal.pone.0151367 (PMC4788447; doi:10.1371/journal.pone.0151367)
Supplement: S9 Fig — The different types of NHEJ repair events were classified as having no microhomology (no MH), having insertions, having microhomology (MH) or other (not shown). The alignments show the flanking region of the break site with are different features highlighted: Insertions (blue) and MH (yellow). The deleted region is represented by the light blue sequences within the brackets. The size of the deletion is shown on the right in red. (PDF) [file pone.0151367.s009.pdf]

Alignment of NHEJ events

NHEJ no MH

Deleted region

Clone	Left flank	Deleted region	Right flank	Deletion
Cd39	GGCAGCAGAATTGCG-	[tgaagccg--------------------aaaaaa]	-AAAGAATTCTTTTCTGCA	1549 deletion
Cd40	GGCGGTTCACGAGGT-	[gaggagat--------------------agactc]	-CGTCTCAAAAAAAAAAAA	1659 deletion
Cd41	AAAAGCAGGCTTGG-	[taccgaga--------------------caaaaa]	-AAAAAAAAAAAGAATTCT	1757 deletion
Cd55	CTGAAACCCTGTCTC-	[tactataa--------------------tcaaaa]	-AAAAAAAAAAAAAAATTC	1626 deletion
Ni45	AAAGCAGGCTTGGTA-	[ccgagaag--------------------ataggg]	-ATAACAGGGTAATAATAT	1410 deletion
Ni48	GGAGACCCAAGCTGG-	[ctaggtaa--------------------ataggg]	-ATAACAGGGTAATAATAT	1068 deletion
Ni50	CTGCAGATATCAACA-	[agtttgta--------------------ataacg]	-GGGTAATAATATTTTTAA	1443 deletion
Ni57	CTGAAAAAAATAAAA-	[aaaaaagg--------------------agggat]	-CAGGGTAATAATATTTTT	1108 deletion
Ni58	ACTATAGGGAGACCC-	[aagctggc--------------------aggtca]	-GGAGATCGAGACAATCCT	1641 deletion
Ni68	AAAAGCAGGCT-	[tggtaccg--------------------ctaggg]	-ATAACAGGGTAATATAG	1332 deletion
Ni70	GTGATCGCACCACTG-	[gaactcca--------------------taggga]	-TAACAGGGTAATATAG	1068 deletion

NHEJ insertions

Untemplated insertions highlighted in blue

Deleted region

Clone	Left flank	Insertion	Deleted region	Right flank	Deletion
Cd42	GCCGCGGAGGCTCAGG	GTAA	[cctgttat----------cagggtaa]	-TAATATTTTTAAACTTGCT	1384 deletion
Cd46	GCCTGAAGTCCCAGC	AAATAT	[tactcagg----------tttttaac]	-TTGCTCTTCAACTGTTGG	1253 deletion
Cd47	AGGCCGGGCGTGGTGG	TAACAATAA	[gtcaggcc----------cagtgagc]	-CGAGATCGCGCCACTG	1668 deletion
Cd51	CAGGCTTGGTACCGAG	GGTAACAGGTACC	[aagcttaa----------cgtctcaa]	-AAAAAAAAAAAAAAGAATT	1745 deletion
Cd52	TACAAAAAAGCAGGCT	AATAT	[tggtaccg----------aggataac]	-AGGGTAATATAGGACCCCA	1336 deletion
Cd53	GGACAGAGCGTGACT	AGAGGTA	[ccgtcaca----------gcataggg]	-ATAACAGGGTAATAATATT	1123 deletion
Ni53	ATAGGGAGACCCAAGC	GGCACCCAAGCGGCACACTATATACGCTGGC	[tggctagg----------aaatagca]	-TAGGGATAACAGGGTAATA	1503 deletion
Ni55	GCTTAAAGGCCGGCCG	TGAT	[cggaggct----------aaaaaaaa]	-AAAAGAATTCTTTTCTGCA	1739 deletion
Ni59	GCGGAGGCTCAGGCCT	TCTGCCTATTCTTA	[gttatccc----------tagcatag]	-GGATAACAGGGTAATAATA	1371 deletion
Ni60	CGAGAAGCTTAAAGGC	TT	[cgggcgcg----------ctcttcaa]	-CTGTTGGATCCACTTAAA	1431 deletion
Ni61	CTGCAGCCTGGGGGA	TTCTAT	[cagagcgt----------cgctaggg]	-ATAACAGGGTAATATAGGA	1051 deletion
Ni62	GCCGGGCGCGGTGGCT	TAA	[caggcctg----------aacccggg]	-AGGCGGAGCTTGCAGT	1646 deletion
Ni63	CTCGGATCCACTAGTC	ATCATAT	[cagtgtgg----------gggaggcg]	-GAGCTTGCAGTGAGCCGAG	1739 deletion
Ni64	TACCGAGAAGCTTAAA	TCT	[ggccggcc----------gcaggcgg]	-CCGCGACACCATGACCGAG	1773 deletion
Ni65	CGGAGGCTCAGGCCTGT	TTA	[tatcccca----------atagggat]	-AACAGGGTAATAATATTTT	1413 deletion
Ni66	ACAAAAAAGCAGGCTT	TTTGTAT	[ggtaccga----------cgctaggg]	-ATAACAGGGTAATATAGGA	1330 deletion
Ni71	TGGTACCGAGAAGCTT	TTCTGCGGGCGGTA	[aaaggccg----------aagaattc]	-TTTTCTGCAGGCGACCG	1761 deletion
As12	CCGGGCGCGGTGGCTC	TA	[aggcctgt----------atcgcgcc]	-ACTGCACTCCAGCCTG	1677 deletion
As13	GATCCACTAGTCCAGT	AT	[ggtggaat----------gctactcg]	-GGAGGCTGAGGGCAGGAGA	1728 deletion
As15	CTATAGGGAGACCCAA	TAGTG	[gctggcta----------atttcttt]	-TCTGCAGGCGGCCGCGACA	1874 deletion

NHEJ MH

Microhomology highlighted in yellow

		Deleted region		
Cd43	TGTCTCACTATAAA-	[tacaaata---------tcaaaaaa]	-AAAAAAAAAAAAAATTCTT	1617 deletion
Cd44	CAGGCTTGGTACCGAG-	[aagcttaa---------cccgggag]	-GCGGAGCTTGCAGTGA	1674 deletion
Cd45	CTGAAACCCTGT-	[ctctact----------caaaaaat]	-TAGCCGGGCGTGGTGGC	1476 deletion
Cd48	AATTCCGTGGAGCCG-	[ggaggcgg---------gtgagccg]	-AGATCGCGCCACTG	1478 deletion
Cd49	AAGTTTGTACAAAAAA-	[gcaggctt----------aaaaaaa]-	AAAAGAATTCTTTTCTGC	1773 deletion
Cd50	ACAAAAAAAAAAAAAA-	[aaaaggcc----------aaaaaaa]-	AGAATTCTTTTCTGCAGG	1455 deletion
Cd54	GGAGGCGGGCGGTTCA-	[cgaggaca---------ccgtctca]	-AAAAAAAAAAAAAAAAAT	1672 deletion
Ni43	AAGTTTGTACAAAAAA-	[gcaggctt---------aaaaaaaa]	-AAAGAATTCTTTTCTGCA	1779 deletion
Ni44	CTGAGGCAGGAGAATT-	[gcgagaag---------aaagaatt]	-CTTTTCTGCAGGCGGCCG	1562 deletion
Ni46	GGCGCGGTGGCTCAGG-	[cctgtaat---------ataacagg]	-GTAATAATATTTTTAAAC	1383 deletion
Ni47	TACCGAGAAGCTTAAA-	[ggccgggc---------ctcttcaa]	-CTGTTGGATCCACTTAAA	1434 deletion
Ni49	GACTCACTATAGGGAG-	[acccaagc---------ctcgggag]	-GCTGAGGCAGGAGAATTG	1753 deletion
Ni51	GTACCGAGAAGCTTAA-	[aggccggg-----------cctgta]	-GTCCCAGCTACTCGGGA	1618 deletion
Ni52	TGTACAAAAAAGCAGG-	[cttggtac---------ggataacg]	-GTAATAATATTTTTAAAC	1425 deletion
Ni54	TATAGGGAGACCCAAG-	[ctggctag---------ttgcagtg]	-AGCCGAGATCGCGCCACT	1793 deletion
Ni56	ACAAAAAAGCAGGCTT-	[ggtaccga---------ctatttat]	-AACAGGGTAATATAGGAC	1333 deletion
Ni69	GGTACCGAGAAGCTTA-	[aaggccgg-----------ggataa]	-CAGGGTAATA	1404 deletion
As11	ACCGAGAAGCTTAAAG-	[gccgggcc---------aaaattag]	-CCGGGCGTGGTGGTGCGG	1589 deletion
As14	TCAACAAGTTTGTACA-	[aaaaagca-----------acccca]	-TTGGTTTAAAAGGCCAAT	1368 deletion

Supplemental Figure S9. Sequence alignments of the break site flanking sequences of the different type of events repaired through non-homologous end joining after metal treatments. The different types of NHEJ repair events were classified as having no microhomology (no MH), having insertions, having microhomology (MH) or other (not shown). The alignments show the flanking region of the break site with are different features highlighted: Insertions (blue) and MH (yellow). The deleted region is represented by the light blue sequences within the brackets. The size of the deletion is shown on the right in red.
